# Optimizing Readability and Format of Plain Language Summaries for Medical Research Articles: Cross-sectional Survey Study

**DOI:** 10.2196/22122

**Published:** 2022-01-11

**Authors:** Leia Martínez Silvagnoli, Caroline Shepherd, James Pritchett, Jason Gardner

**Affiliations:** 1 Department of Life Sciences Faculty of Science and Engineering Manchester Metropolitan University Manchester United Kingdom; 2 CMC Connect McCann Health Medical Communications Macclesfield United Kingdom

**Keywords:** biomedical research, health literacy, multiple sclerosis, plain language summary, psoriasis, rheumatoid arthritis

## Abstract

**Background:**

Plain language summaries (PLSs) are intended to provide readers with a clear, nontechnical, and easily understandable overview of medical and scientific literature; however, audience preferences for specific PLS formats have yet to be fully explored.

**Objective:**

This study aims to evaluate the preferred readability level and format for PLSs of medical research articles of different disease states via a web-based survey of audiences of different age groups.

**Methods:**

Articles describing phase III clinical trials published in top-level, peer-reviewed journals between May 2016 and May 2018 were identified for 3 chronic disease states representing a range of adult patient age groups: (1) psoriasis, a skin disease representative of younger patients; (2) multiple sclerosis (MS), a neurological disease representative of middle-aged patients; and (3) rheumatoid arthritis (RA), a painful joint disease representative of older patients. Four PLSs were developed for each research article, of which 3 were text-only summaries (written with high, medium, and low complexity) and 1 was an infographic. To evaluate each of the 4 PLS formats, a 20-question open survey (specific to one of the 3 diseases) was sent to a representative sample selected via UK-based patient association websites, Twitter, and Facebook patient groups. A weighted-average calculation was applied to respondents’ ranked preferences for each PLS format.

**Results:**

For all 3 articles, the weighted-average preference scores showed that infographic (psoriasis 2.91, MS 2.71, and RA 2.78) and medium-complexity text-based PLS (reading age 14-17 years, US Grade 9-11; psoriasis 2.90; MS 2.47; RA 2.77) were the two most preferred PLS formats.

**Conclusions:**

Audience preferences should be accounted for when preparing PLSs to accompany peer-reviewed original research articles. Oversimplified text can be viewed negatively, and graphical summaries or medium-complexity text-based summaries appear to be the most popular.

**Plain Language Summary:**

Patients and caregivers should have the chance to read about medical research in a format they can understand. However, we do not know much about the formats that people with different illnesses or ages prefer. Researchers wanted to find out more about this. They selected 3 medical articles about illnesses that affect different age groups: psoriasis (younger patients), multiple sclerosis (middle-aged patients), and rheumatoid arthritis (older patients). They created 4 summaries of each article. One was a graphical summary, and the other 3 were words-only summaries of high, medium, and low complexity. Then, the researchers posted surveys on UK patient group websites and Facebook patient groups to ask people what they thought of the summaries. The surveys were taken by 167 people. These people were patients with psoriasis, multiple sclerosis, or rheumatoid arthritis, or their caregivers. Most were women, and about half had a university degree. For each illness, most people preferred the graphical summary. Among the word-only summaries, most people preferred the medium-complexity wording written for a reading age of 14 to 17 years. People felt that the graphical and medium-complexity summaries were clear and concise, while the others used jargon or were too simple. Authors of medical articles should remember these results when writing summaries for patients. More research is needed about the preferences of other people, such as those with other illnesses. (See Multimedia Appendix 1 for the graphical summary of the plain language summary.)

## Introduction

### Background

Health literacy, that is, the degree to which individuals can obtain, process, and understand basic health information to make appropriate health decisions [1,2], is critical to the patient-doctor relationship [3]. Health information should be easy to access, use, and understand for everyone, including both patients and their caregivers. However, despite the increasing availability of medical content from different forms of media, studies have shown that few nonexperts can understand, or act on, the health information available [2] and that text is often written above the general readability level, in a way that limits understanding and hinders the ability to make informed choices [3,4]. Indeed, in a 2019 survey of more than 14,000 people in the United States, 88% of respondents thought that “scientists should be sharing their results in easy-to-understand language” [5].

### Plain Language Summaries

Plain language summaries (PLSs) have been introduced to make written and verbal information more easily understood by nonexperts [6-8]. Such strategies are gradually being adopted across all documents, presentations, and electronic communications intended for the public to avoid the use of jargon and highly technical language, and to focus on the information that is most relevant for patients, caregivers, and families [1,7]. Text is written in an easily readable style with short, clear sentences, using everyday English words, and avoiding complex grammatical structures wherever possible [6]. Thus, a PLS can explain complicated medical research to the nonexpert, thereby extending the reach of scientific information and empowering nonexperts with the knowledge to act on the information they receive [7-10].

Through the use of PLSs, scientific information is given a direct route from researchers to a broader audience beyond the scientific community. PLSs provide greater clarity to all those interested in learning about expert scientific material [11], while reducing the risk of overinterpretation via journalism or social media [12]. It is important to recognize that PLSs can be for everyone—from nonspecialists, including patients, caregivers, the lay public, and nonexperts in the field of research [1,6], to busy medical specialists and other healthcare providers [6,12]. For healthcare professionals, establishing new standards of communication, such as PLS, will improve their ability to meet the needs of quickly changing health systems and increasingly globalized populations [13]. Furthermore, a wide distribution of information is also expected to improve patient and healthcare provider engagement [1,2,10,13], promoting an increased focus on disease research and public support. Many research organizations now have public blogs on their websites, which discuss certain aspects of their research that may not necessarily be covered by scientific publications [11]. Encouraging public involvement in this way can improve the quality of research and also help with the development of new research strategies [11].

PLS is a term used to cover many forms of summary information in the medical or scientific setting. It is important to make the distinction between two of the most common forms of PLS, as explained below.

### Types of PLS

The first is a clinical trial summary (CTS), where clinical trial sponsors produce a brief summary of the trial, focusing on the main results (ie, the primary endpoint and key safety data). These summary documents are shared with trial participants and the general public; they are usually posted on the sponsor’s website or an independent electronic repository. CTS are a mandatory requirement of the European Union Clinical Trials Regulation and Good Lay Summary Practice (GLSP) recommendations have recently been published as part of the EudraLex Volume 10 clinical trials guidelines [14]; for the United States, a draft guidance document making similar CTS recommendations was submitted to the FDA in 2017 [1,15,16]. The elements that must be contained within a CTS are strictly defined within these regulatory guidelines. CTSs are not the subject of our research.

The second form of PLSs, which is the focus of our research, relates to summarizing a peer-reviewed article published in a medical journal [9,12]. These PLSs act as easy-to-read executive summaries of the most up-to-date research published in the medical literature. They are usually optional and published as a free, open-access document alongside the associated medical journal article. Hereafter we refer to a PLS in the context of summarizing the medical literature.

### Development of PLSs

The benefits of PLSs have been recognized [17]; previous studies have aimed to understand different stakeholders’ perspectives on PLSs [18]; and helpful tools are available to assist with the development of PLSs [6,19-21]. However, research on the most effective communication strategies remains limited. For example, it is unclear whether most audiences prefer text-based articles or more visual formats using infographics (ie, graphs and charts that provide clear information) [8,22,23]. Crick and Hartling [23] found that doctors preferred PLSs in text format, whereas nurses preferred an infographic format. Buljan et al [9] found that students, doctors, and consumers (female members of a patient and parent action group) reported no difference in the knowledge they obtained from infographic or text-based PLSs. Therefore, although these studies offer interesting insights, there is little evidence regarding the preferred format of PLSs of publications read by lay audiences, considering populations representative of those seeking information from the medical literature.

Different text readability formulas are available to aid the development of PLSs [24-26], but the level of complexity that should be applied to text-based PLSs remains to be established. A survey of the adult general population in England indicated that approximately half the population has only basic literacy skills, of General Certificate of Secondary Education (GCSE) Grade D and below [27]. The UK Government Digital Service suggests that content should be developed to reflect the reading age of a 9-year-old child [28]. Furthermore, expert group recommendations for CTSs of European registered clinical trials state that these summaries should normally be accessible by young people from the age of 12 years of age and above, and that sponsors should consider testing CTS readability among those representing the target population [15]. However, it remains to be determined which literacy level(s) should be considered when developing PLSs of medical literature and whether this would differ with topic (eg, disease type) and the age range of the target reader.

### Study Aim

This cross-sectional study aims to evaluate the preferred readability and format for PLSs of medical research pertaining to chronic diseases affecting different age ranges, among web-based, lay audiences (ie, patients and caregivers) who may likely have an interest in obtaining information about the latest research in the field.

## Methods

Three chronic diseases were chosen, representing different age band classifications based on the age groups commonly affected by these conditions—psoriasis, representative of a predominantly younger population; multiple sclerosis (MS), representative of a predominantly middle age group; and rheumatoid arthritis (RA), representative of predominantly older patients. To source relevant articles, journals were selected based on their impact factor and narrowed down to those that published research articles focusing on all 3 diseases. Specific articles were identified using the PubMed database, searching for randomized controlled phase III trials published from May 2016 to May 2018.

**Figure 1:**
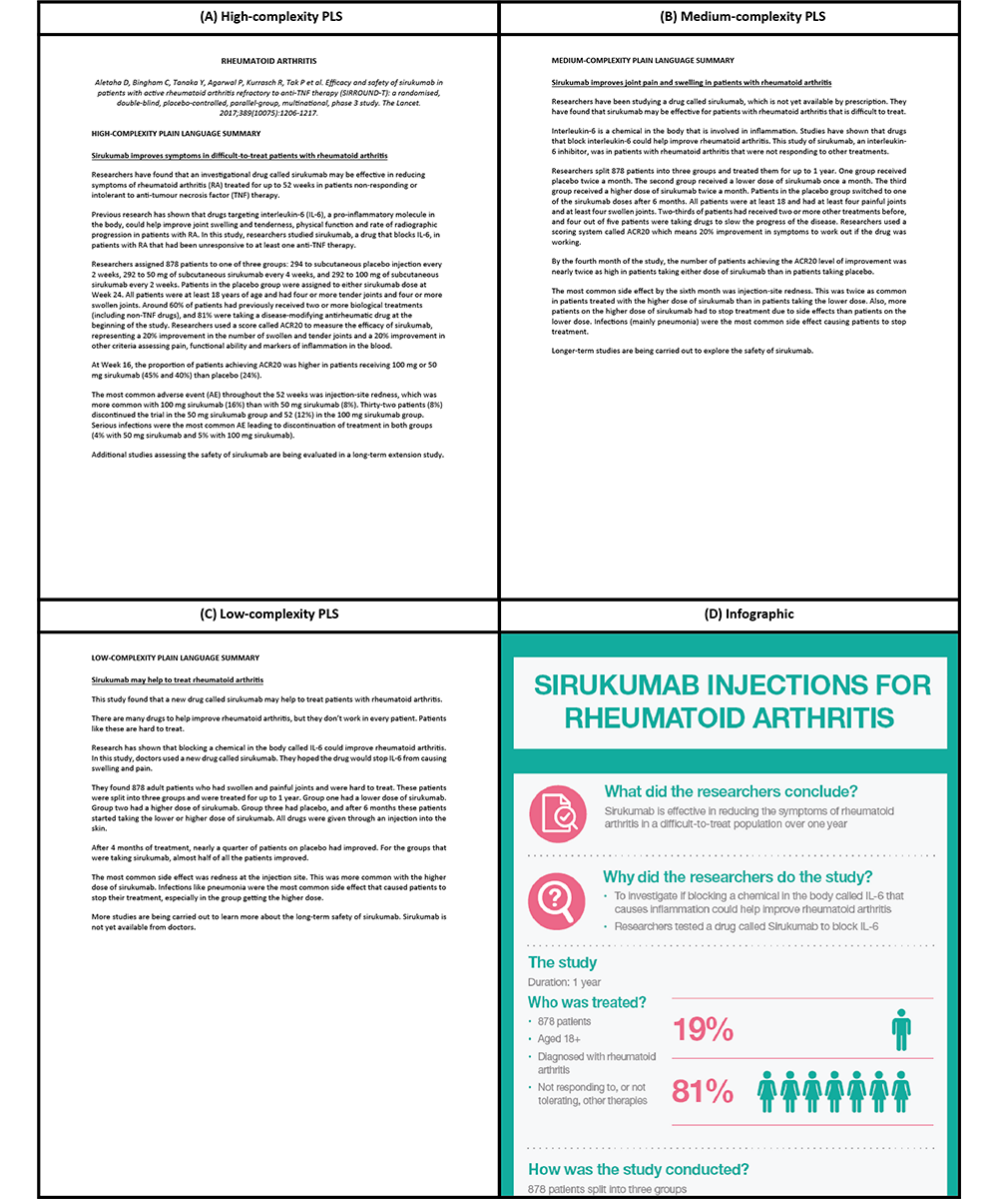
Examples of various PLS formats used. (A) High-complexity text-only PLS, (B) medium-complexity text-only PLS, (C) low-complexity text-only PLS, and (D) infographic PLS format. Text complexity in each case was determined using an automated readability checker from Readabilityformulas website [24], using text from the main body only (ie, excluding title, authors, and funding statements) and omitting any parenthetical data. Full versions of the infographics analyzed are shown in Multimedia Appendices 1-3. PLS: plain language summary.

One article was selected for each chronic disease [29-31]. Four PLSs were developed for each of the 3 articles; of these, 3 PLSs were text-only summaries (ie, written with high, medium, and low levels of complexity) and the fourth PLS was an infographic (see Figure 1 and Multimedia Appendices 2, 3, 4). Complexity of the text (based on main body text only) was determined using an automated readability checker from the Readabilityformulas website [24]. Varying levels of complexity (ie, high, medium, and low) for the text-only PLSs were measured and adapted by changing variables such as the length and number of sentences, syllable count, and use of acronyms (a summary of the differences in text complexity for each PLS vs the abstract of each original article is provided in Multimedia Appendix 5).

A 20-question (1 question per page), web-based open survey was developed using SurveyMonkey [12] to assess the readability of, and preference for, each of the 4 PLS formats (Multimedia Appendix 6). The order of presentation of the PLSs to the survey respondents was as follows: (1) high-complexity text-only PLS, (2) medium-complexity text-only PLS, (3) low-complexity text-only PLS, and (4) infographic format. The usability and technical functionality of the survey were tested by one of the authors’ colleagues who had no scientific qualifications. The survey was sent to organizations representing patients and caregivers for each of the 3 conditions. The survey was accessed via UK-based patient association websites and Facebook patient support groups (Multimedia Appendix 7). Specific associations and patient support groups approached for this study include Psoriasis and Psoriatic Arthritis Alliance, Psoriasis Association, Psoriasis Support Group UK, MS Society, Multiple Sclerosis Trust, MS-UK, Asian MS (UK national support group), Multiple Sclerosis Support/Chat Group UK, Mutual Support (Armed Forces), National Rheumatoid Arthritis Society Regional groups, Arthritis Research UK, Arthritis Care (part of Arthritis Research UK), Arthritis Action, and the UK Rheumatoid Arthritis Wonky Group. Each organization chose its wording to advertise the survey, based on the background information provided. Participation in the survey was voluntary, and questions could be skipped (no nonresponse options were provided). Respondents could move back and forward throughout the survey to review and change their answers before submission. No incentives to complete the survey were provided. The survey was active for 3 weeks, between August 10 and September 2, 2018.

Ethical approval was obtained from the Manchester Metropolitan University Research Ethics Committee. All survey responses were anonymous, and no personal information or identifying information were collected or made available to the researchers.

Participants were informed of the scholarly purpose of the study, details of the principal investigator, estimated length of time for survey completion, and anonymity of data they were to provide. Cookies inherent to the SurveyMonkey platform were used, which prevented duplicate entries [32]. Some data were not collected, including any assessment of unique site visitors, view rate (ie, ratio of unique survey visitors or unique site visitors), and participation rate (ie, ratio of unique visitors who agreed to participate or unique first survey page visitors).

The completion rate was determined by calculating the ratio of total number of respondents who finished the survey to total number of respondents who initiated the survey. All data were included in the analysis, regardless of whether the survey was fully completed.

No formal statistical analyses were performed on these data. A weighted-average calculation, performed through the SurveyMonkey platform, determined the average ranking for each PLS option to identify the most preferred format. The format with the highest average ranking score indicates the respondents’ preferred option.

The average ranking was calculated as follows:




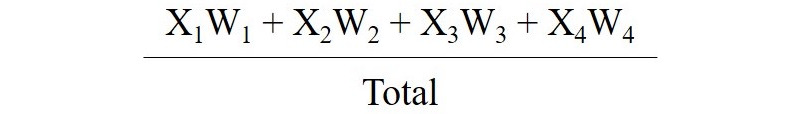




where *w* is the weight of the ranked position, and *x* is the response count for the corresponding answer choice.

For each person who responded, the most preferred choice (ranked as #1) was assigned the largest weight (in this case: 4); by contrast, the least preferred choice (ranked #4) was assigned a weight of 1. No data adjustments were made.

Subgroup analyses were also performed to identify PLS preference based on individuals’ age (younger, 18-34 years; middle-aged, 35-54 years; or older, ≥55 years), gender (female, male, or other), and education level (nondegree level or university degree level, defined as including a UK university bachelor’s degree, master’s degree, PhD, or other postgraduate degree).

This article was prepared in accordance with the Checklist for Reporting Results of Internet E-Surveys (CHERRIES; see Multimedia Appendix 1) [33].

## Results

### Survey Respondents

**Figure 2:**
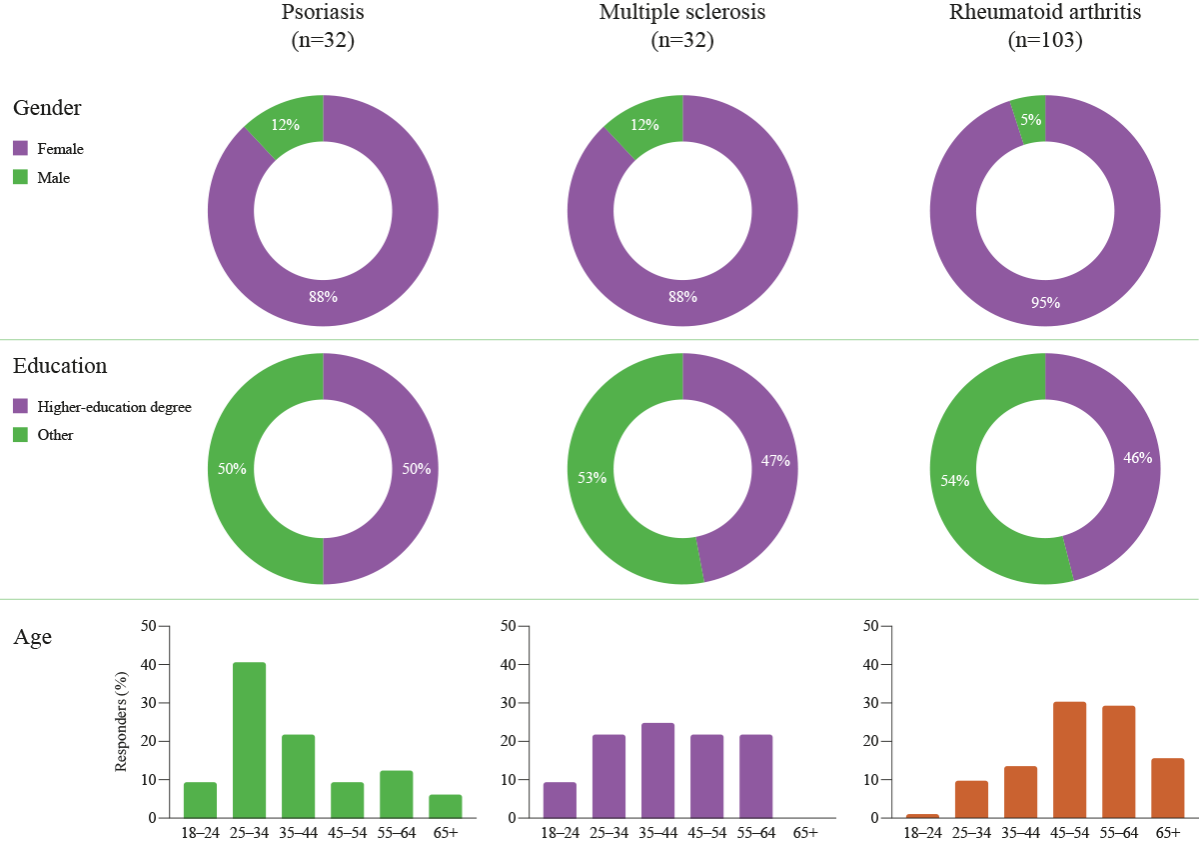
Demographics of survey respondents across different chronic disease states.

In total, 167 survey responses were received for the 3 surveys (psoriasis, n=32; MS, n=32; RA, n=103; Figure 2). The survey completion rates were 84% (27/32) for psoriasis, 81% (26/32) for MS, and 90% (93/103) for RA. Those who responded to the survey were mainly women (psoriasis, 28/32, 88%; MS, 28/32, 88%; RA, 97/102, 95%), and approximately half were educated to university (higher-education) degree level (psoriasis, 16/32, 50%; MS, 15/32, and 47%; RA, 47/102, 46%). Age ranges for respondents were as expected for each of the 3 disease states.

### Primary Analysis

**Figure 3:**
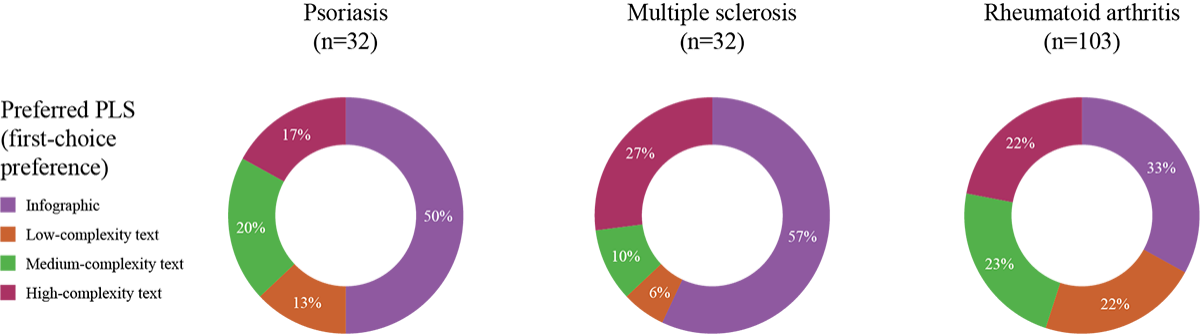
Respondents’ first-choice preference of PLS format for the different disease states. PLS: plain language summary.

Across all 3 disease states, the infographic was the first-choice PLS format for most respondents (psoriasis, 15/30, 50%; MS, 17/30, 57%; RA, 33/100, 33%), whereas the low-complexity text-only PLS was the least preferred first-choice format (psoriasis, 4/30, 13%; MS, 2/30, 6%; RA, 22/100, 22%; see Figure 3).

**Figure 4:**
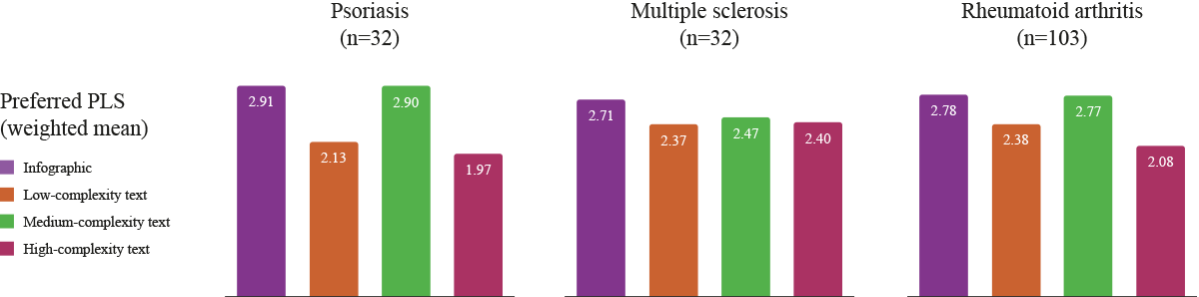
Weighted mean scores for preferred PLS format. PLS: plain language summary.

Similarly, results from the weighted-average preference score data demonstrated that the infographic (psoriasis 2.91; MS 2.71; RA 2.78) and medium-complexity text-only PLSs (reading age 14-17 years, US Grade 9-11; psoriasis 2.90; MS 2.47; RA 2.77) were the 2 most popular PLS formats across all 3 diseases analyzed (Figure 4).

### Subgroup Analysis

Among those who chose the infographic format as their first-choice preference, the majority for both the psoriasis and MS groups were in the younger age category of 18 to 34 years (psoriasis 10/15, 67% and MS 8/17, 47%). In contrast, for the RA group, the preference for the infographic format was similar among middle-aged (35-54 years; 16/33, 49%) and older respondents (≥55 years; 14/33, 42%; see Multimedia Appendix 8). When analyzed by education level, we found that about half the respondents who preferred the infographic format for psoriasis and MS had a higher-education degree (psoriasis 8/15, 53% and MS 8/17, 47%); this proportion was lower for RA (12/33, 36%; see Multimedia Appendix 8).

Of the respondents who preferred the medium-complexity text-only PLS format, the majority in each group were ≥55 years of age for MS (2/4, 50%) and RA (14/23, 61%); however, for psoriasis, the preference was equal across all 3 age groups analyzed (2/6, 33%; see Multimedia Appendix 8). The proportion of respondents with a higher-education degree and those who preferred the medium-complexity text-only PLS was similar for psoriasis (3/6, 50%) and MS (2/4, 50%), but higher for RA (14/23, 61%).

### Free-text Feedback Samples

As part of the survey, respondents were able to provide free-text feedback regarding the different PLS formats (Multimedia Appendix 9). Responses were almost the same across the 3 disease types. In general, infographic and medium-complexity text-only PLS formats were praised for their clear and concise presentation, while maintaining the relevant level of information. In contrast, the high-complexity and low-complexity text-only PLS formats were criticized for the use of jargon or oversimplification, respectively.

Reasons (verbatim) provided by the survey respondents for specific preference for the infographic or medium-complexity text-only PLS formats are listed below:

…[infographic] helpful in getting statistical information across.

...[infographic] clear, concise, and easy to understand.

…graphic summary was accessible almost at a glance.

…[infographic] well detailed and a lot easier to read than loads of text.

[medium-complexity] summary combined straightforward language with enough information.

[medium-complexity] text was well detailed and in a language that was easy to understand by anyone.

[medium-complexity] would ‘fill-in-the-gaps’ and provide the detail that the [infographic] summary by its very nature could not.

Reasons (verbatim) provided by the survey respondents that suggest the high-complexity text-only and low-complexity text-only PLS formats were less popular are listed below:

The [high-complexity text] was way too hard to understand what they were saying, too many big words.

[High-complexity] summary was quite complex to understand.

[High-complexity] requires greater concentration and previous experience of medical terminology, e.g., AE – not all would know that this means adverse event.

I ranked the [low-complexity] text summary 4th because, although it was short and easy to read, it did not give me the pertinent statistical data from which the conclusion was drawn.

I thought the [low-complexity] text summary had been simplified to the extent that it lost some meaning.

I personally hate dumbed-down items… Difficult subjects should be explained…

## Discussion

Our findings showed a clear preference for an infographic PLS format among the 3 disease states we assessed (psoriasis, MS, and RA). Medium-complexity (reading age 14-17 years, US Grade 9-11) was the most preferred text-based format. The main reasons cited for preferring these formats were that the information presented was clear, concise, easy to understand, and included relevant detail, without oversimplification of the content. The majority of respondents were women, and approximately half had a university-level degree. Preferences remained the same regardless of education status; however, younger respondents were more likely to prefer the infographic to the text-based format.

PLSs are an important tool for improving health literacy. Research has indicated that most nonexperts express difficulty understanding medical and scientific texts, especially when reading text describing complex clinical research [34].

In recent years, there have been initiatives to improve the reach of information meant for a nonexpert audience. In 2010, the United States Congress recognized the need for the use of plain language when communicating information intended for the public [35]. For example, The National Action Plan to Improve Health Literacy aims to engage organizations, professionals, policy makers, communities, individuals, and families in a linked, multi-sector effort to improve the understanding of basic health information [36]. The plan is based on two “core principles”: (1) all people have the right to health information that helps them make informed decisions, and (2) health information should be delivered in ways that are easy to understand and that improve health, longevity, and quality of life. Moreover, the US National Institutes of Health aim to broaden the reach of health information to all Americans by communicating research results in terms that are easy to understand [7]. Similar initiatives are taking place in the European Union, where CTS must accompany clinical trial results for laypersons [15]. CTSs aim to be accessible to the general public as young as 12 years [15]. Health literacy is important to young patients; therefore, supporting them in understanding health issues can empower them to take control of their health and provide the information they need to seek appropriate services [37].

Additionally, in 2017, to increase public access to healthcare information, the nonprofit organization eLife compiled a list of organizations that provide PLSs of published scientific research—including more than 50 medical or scientific journals [38]. We now estimate that more than 250 medical journals now facilitate the provision of PLSs; however, wide variations still exist in the terms used for defining a PLS, the presentation format, the platforms where they are located (eg, journal website, Figshare, or Kudos), and how the reader may discover them (eg, via a PubMed search) [39].

To standardize PLS formats, readability scores and formulas (within applications like Microsoft Word or by using web-based tools [24]) have been used to assess the complexity of text; however, such a metrics-based approach fails to incorporate individual preferences regarding information delivery and overlooks the importance of engaging the audience or assessing whether the information will be interpreted as intended. Furthermore, although PLS formats are far easier to read than other traditional formats, the level of literacy preferred by the population surveyed in our study was relatively higher than the recommended reading age for a CTS and health-related information (generally 10-12 years of age, US Grade 5-7) [4,15,40,41].

The best way of presenting research results to different audiences remains unclear. Few studies have investigated this topic, which indicates that scientific findings can be difficult to interpret [9]. Furthermore, other research has identified that, in addition to PLS, video abstracts may be preferable than published text or graphical abstracts [42]. Our study provides valuable insight and direction for how PLSs may be formatted and presented to communicate original medicine-based research to a broader audience. Through the use of a survey, people who responded were able to state their preferences for PLS formats and also provide reasons for their preference, referring to the key factors that dictate how they wish to receive scientific information. The participants’ preference for both the infographic and medium-complexity text-based PLS format was based on clarity and concise distribution of information, without sacrificing key details. The high-complexity text-based PLS was thought to have excessive use of jargon, requiring a scientific background to appreciate the information adequately. Conversely, participants were dissatisfied with the low-complexity PLS format, considering it too simple and not having enough substance.

### Limitations

The overall sample may have been a more educated population than the general public, being sourced from patient groups and those who regularly use the internet and social media. We consider this more educated population to be representative of the technology-competent, information-seeking individuals most likely to be sourcing and reading PLSs; however, it does not necessarily capture the preferences of audiences who are less technologically aware and who may still benefit from exposure to clinical research through reading PLSs.

There was a notable gender imbalance within all 3 subpopulations surveyed, with approximately 90% of the respondents being women in each case. Women are more likely to experience RA and MS than men, whereas the prevalence is about equal for those with psoriasis [43-45]; however, the imbalance was far more significant in this survey than that observed in the real-world setting, which we are unable to explain. The survey also did not capture whether those who responded were patients or caregivers, which could have provided more context to the results.

Although the samples for each disease included in the survey provided enough data to generate meaningful results, the number of people who responded to the psoriasis and MS surveys were only a third of those who responded to the RA survey. Results of the subgroup analyses should be treated with caution due to the low number of respondents in each subgroup, particularly from the psoriasis and MS populations.

Since developing the PLS for each of the 3 source articles surveyed, our understanding and application of best practices in plain language writing for publications have advanced. If we were to repeat this project, we would apply more principles outlined in the tools to help guide plain language writing [6,14,18-20] to the development of PLSs used in the analyses.

Furthermore, although we used a web-based tool to assess readability [24], we recommend caution when using such tools for content that is confidential and where the security of the tool has not been verified.

### Conclusions

Audience preferences should be accounted for when preparing a PLS to supplement an original peer-reviewed research article. However, oversimplification of text can be viewed negatively, and infographic versions or medium-complexity text appear to be the most popular. Further research would be useful to expand both the scope of the therapy areas covered and the profile of those surveyed to include other nonexpert populations and healthcare professionals from other fields of study. It would also be of interest to evaluate the understanding of the information presented in a PLS rather than focus on the preferred format alone. Training at professional societies such as the International Society for Medical Publication Professionals [46] and the widespread use of additional tools now available to guide the effective production of PLSs [6,14,19-21] will help facilitate this.

## Appendix

Multimedia Appendix 1 Plain language summary of this article in an infographic format.

Multimedia Appendix 2 High-, medium-, and low-complexity text-based plain language summaries for psoriasis.

Multimedia Appendix 3 High-, medium-, and low-complexity text-based plain language summaries for multiple sclerosis.

Multimedia Appendix 4 High-, medium-, and low-complexity text-based plain language summaries for rheumatoid arthritis.

Multimedia Appendix 5 Summary of readability scores and other features of text-based plain language summaries for all 3 sample articles evaluated. PLS: plain language summary.

Multimedia Appendix 6 Survey questionnaire for all 3 disease states evaluated.

Multimedia Appendix 7 UK-based patient associations and Facebook groups used to identify survey respondents.

Multimedia Appendix 8 Subgroup analyses. MS: multiple sclerosis; PLS: plain language summary; RA: rheumatoid arthritis.

Multimedia Appendix 9 Free-text feedback on reasons for the preferred PLS format. PLS: plain language summary.

Multimedia Appendix 10 Checklist for Reporting Results of Internet E-Surveys (CHERRIES).

## Abbreviations

CHERRIES: Checklist for Reporting Results of Internet E-Surveys
CTS: clinical trial summary
GCSE: General Certificate of Secondary Education
GLSP: Good Lay Summary Practice
MS: multiple sclerosis
PLS: plain language summary
RA: rheumatoid arthritis

## References

[1] Barnes A, Patrick S (2019). Lay summaries of clinical study results: an overview. Pharmaceut Med.

[2] Stossel LM, Segar N, Gliatto P, Fallar R, Karani R (2012). Readability of patient education materials available at the point of care. J Gen Intern Med.

[3] Koh HK, Brach C, Harris LM, Parchman ML (2013). A proposed 'health literate care model' would constitute a systems approach to improving patients' engagement in care. Health Aff (Millwood).

[4] Karačić J, Dondio P, Buljan I, Hren D, Marušić A (2019). Languages for different health information readers: multitrait-multimethod content analysis of Cochrane systematic reviews textual summary formats. BMC Med Res Methodol.

[5] State of Science Index Survey. 3M.

[6] Duke M (2012). How to write a lay summary. Digital Curation Centre.

[7] (2020). Clear communication. National Institutes of Health – Office of Communications and Public Liaison.

[8] Kerwer M, Chasiotis A, Stricker J, Günther A, Rosman T (2021). Straight from the scientist’s mouth—plain language summaries promote laypeople’s comprehension and knowledge acquisition when reading about individual research findings in psychology. Psychology.

[9] Buljan I, Malički M, Wager E, Puljak L, Hren D, Kellie F, West H, Alfirević Ž, Marušić A (2018). No difference in knowledge obtained from infographic or plain language summary of a Cochrane systematic review: three randomized controlled trials. J Clin Epidemiol.

[10] Pushparajah DS, Manning E, Michels E, Arnaudeau-Bégard C (2018). Value of developing plain language summaries of scientific and clinical articles: a survey of patients and physicians. Ther Innov Regul Sci.

[11] Salita JT (2015). Writing for lay audiences: a challenge for scientists. Medical Writing.

[12] Blyth J, Gaskarth M, Plant A, Woods K (2019). We need to talk about PLS...exploring the opportunities of plain-language summaries. ISMPP Newsletter.

[13] Warde F, Papadakos J, Papadakos T, Rodin D, Salhia M, Giuliani M (2018). Plain language communication as a priority competency for medical professionals in a globalized world. Can Med Educ J.

[14] (2021). Good Lay Summary Practice. Clinical Trials Expert Group.

[15] (2017). Summaries of clinical trials for laypersons. European Commission.

[16] (2017). Plain language summary guidance document submitted to the FDA–open for comments. Multi-regional Clinical Trials Center.

[17] (2021). Plain English summaries: What is a plain English summary?. National Institute for Health Research.

[18] Lobban D, Gardner J, Matheis R Plain language summaries of publications of company-sponsored medical research: what key questions do we need to address?. Curr Med Res Opin. Epub ahead of print posted online on November 4, 2021.

[19] (2018). Plain language summaries (PLS) of publications toolkit: a best-practice resource for PLS of peer-reviewed publications and congress abstracts. Envision Pharma Group.

[20] (2013). Methodological Expectations of Cochrane Intervention Reviews (MECIR): Standards for the reporting of plain language summaries in new Cochrane Intervention Reviews. Cochrane Methods.

[21] Rosenberg A, Baróniková S, Feighery L, Gattrell W, Olsen RE, Watson A, Koder T, Winchester C (2021). Open Pharma recommendations for plain language summaries of peer-reviewed medical journal publications. Curr Med Res Opin.

[22] Chamberlain James L, Bharadia T (2019). Lay summaries and writing for patients: Where are we now and where are we going?. Medical Writing.

[23] Crick K, Hartling L (2015). Preferences of knowledge users for two formats of summarizing results from systematic reviews: infographics and critical appraisals. PLoS One.

[24] (2019). Readability formulas and the active role of the reader. Readability Formulas.

[25] Flesch R (1948). A new readability yardstick. J Appl Psychol.

[26] Kincaid JP, Fishburne R, Rogers R, Chissom B (1975). Derivation of new readability formulas (automated readability index, fog count, and Flesch reading ease formula) for Navy enlisted personnel.

[27] (2011). The 2011 Skills for Life Survey: A Survey of Literacy, Numeracy and ICT Levels in England. Department for Business Innovation and Skills.

[28] (2016). Content design: planning, writing and managing content. UK Government Digital Service.

[29] Warren RB, Mrowietz U, von Kiedrowski R, Niesmann J, Wilsmann-Theis D, Ghoreschi K, Zschocke I, Falk TM, Blödorn-Schlicht N, Reich K (2017). An intensified dosing schedule of subcutaneous methotrexate in patients with moderate to severe plaque-type psoriasis (METOP): a 52 week, multicentre, randomised, double-blind, placebo-controlled, phase 3 trial. Lancet.

[30] Hauser SL, Bar-Or A, Comi G, Giovannoni G, Hartung H, Hemmer B, Lublin F, Montalban X, Rammohan KW, Selmaj K, Traboulsee A, Wolinsky JS, Arnold DL, Klingelschmitt G, Masterman D, Fontoura P, Belachew S, Chin P, Mairon N, Garren H, Kappos L, OPERA IOPERA II Clinical Investigators (2017). Ocrelizumab versus interferon beta-1a in relapsing multiple sclerosis. N Engl J Med.

[31] Aletaha D, Bingham CO, Tanaka Y, Agarwal P, Kurrasch R, Tak PP, Popik S (2017). Efficacy and safety of sirukumab in patients with active rheumatoid arthritis refractory to anti-TNF therapy (SIRROUND-T): a randomised, double-blind, placebo-controlled, parallel-group, multinational, phase 3 study. Lancet.

[32] Survey Monkey About the cookies we use.

[33] Eysenbach G (2004). Improving the quality of web surveys: the Checklist for Reporting Results of Internet E-Surveys (CHERRIES). J Med Internet Res.

[34] Smith CA, Hetzel S, Dalrymple P, Keselman A (2011). Beyond readability: investigating coherence of clinical text for consumers. J Med Internet Res.

[35] United States Congress (2010). Plain Writing Act of 2010. Public Law 111-274-Oct. 13 2010. 124 STAT. 2861. Congressional record, Vol. 156 (2010).

[36] (2010). National action plan to improve health literacy. US Department of Health and Human Services, Office of Disease Prevention and Health Promotion.

[37] Hagell A Association for Young People's Health. Promoting young people's health literacy and understanding their help-seeking behaviour.

[38] (2017). Plain-language summaries: journals and other organizations that produce plain-language summaries. eLIFE.

[39] FitzGibbon H, King K, Piano C, Wilk C, Gaskarth M (2020). Where are biomedical research plain-language summaries?. Health Sci Rep.

[40] Rowlands G, Bond B, Shay M (2013). The Information Standard Workshop: Understanding Health Literacy – best practice in developing and testing health and care information. The Information Standard.

[41] Badarudeen S, Sabharwal S (2010). Assessing readability of patient education materials: current role in orthopaedics. Clin Orthop Relat Res.

[42] Bredbenner K, Simon SM (2019). Video abstracts and plain language summaries are more effective than graphical abstracts and published abstracts. PLoS One.

[43] Humphreys JH, Verstappen SMM, Hyrich KL, Chipping JR, Marshall T, Symmons DPM (2013). The incidence of rheumatoid arthritis in the UK: comparisons using the 2010 ACR/EULAR classification criteria and the 1987 ACR classification criteria. Results from the Norfolk Arthritis Register. Ann Rheum Dis.

[44] Mackenzie IS, Morant SV, Bloomfield GA, MacDonald TM, O'Riordan J (2014). Incidence and prevalence of multiple sclerosis in the UK 1990-2010: a descriptive study in the General Practice Research Database. J Neurol Neurosurg Psychiatry.

[45] Springate DA, Parisi R, Kontopantelis E, Reeves D, Griffiths CEM, Ashcroft DM (2017). Incidence, prevalence and mortality of patients with psoriasis: a U.K. population-based cohort study. Br J Dermatol.

[46] The International Society for Medical Publication Professionals.

