# Supplementary material for: Optimizing Readability and Format of Plain Language Summaries for Medical Research Articles: Cross-sectional Survey Study
Source: J Med Internet Res. 2022 Jan 11;24(1):e22122. doi: 10.2196/22122 (PMC8790687; doi:10.2196/22122)
Supplement: Multimedia Appendix 1 [file jmir_v24i1e22122_app1.pdf]

How do patients prefer to read about medical research?

What did the researchers conclude?

Patients and caregivers looking for online information on psoriasis, multiple sclerosis or rheumatoid arthritis prefer to read about medical research in:

Graphic images

Or text written for people with a reading age of 14-17 years

Why did the researchers do the study?

It's important that patients and caregivers have access to medical research in a language and format that they can understand. But there is not much known about what lay audiences prefer. And whether preference differs for different illnesses and different ages

What was the study design?

The researchers developed summaries of 3 medical articles:

- **Psoriasis (younger patients)**
- **Multiple sclerosis (middle-aged patients)**
- **Rheumatoid arthritis (older patients)**

The researchers wrote 4 summaries for each medical research article:

- **Words only (high complexity)**
- **Words only (medium complexity)**
- **Words only (low complexity)**
- **Graphic format**

There was a separate survey for each illness

20 questions

The survey was posted on UK patient group websites and Facebook patient groups

For example:

psoriasis · MS UK · MS Society · Arthritis Action · nras · Arthritis Research UK · papaa · Multiple Sclerosis Trust · facebook · Arthritis Care

What were the results?

167 people took the survey

Psoriasis 32 people · Multiple sclerosis 32 people · Rheumatoid arthritis 103 people

Most were female

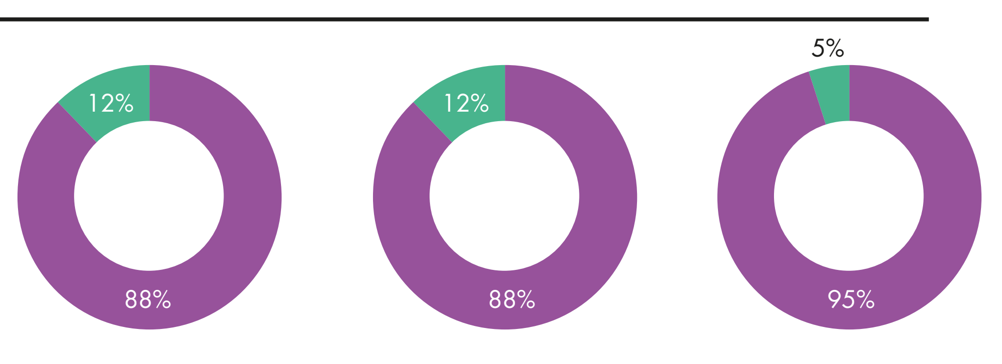

Over half had a university degree

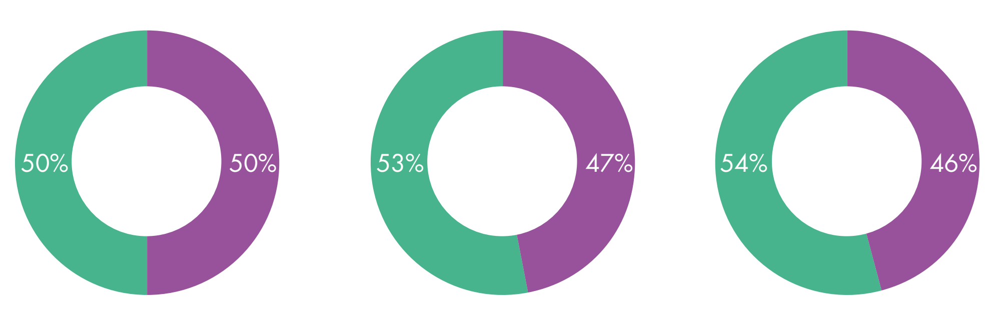

Age ranges matched the three illnesses

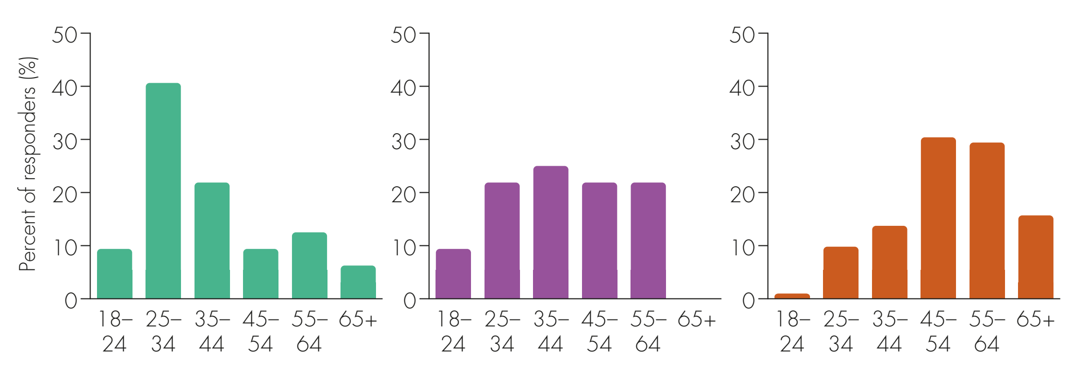

Responders preferred the graphic summaries in all 3 illnesses

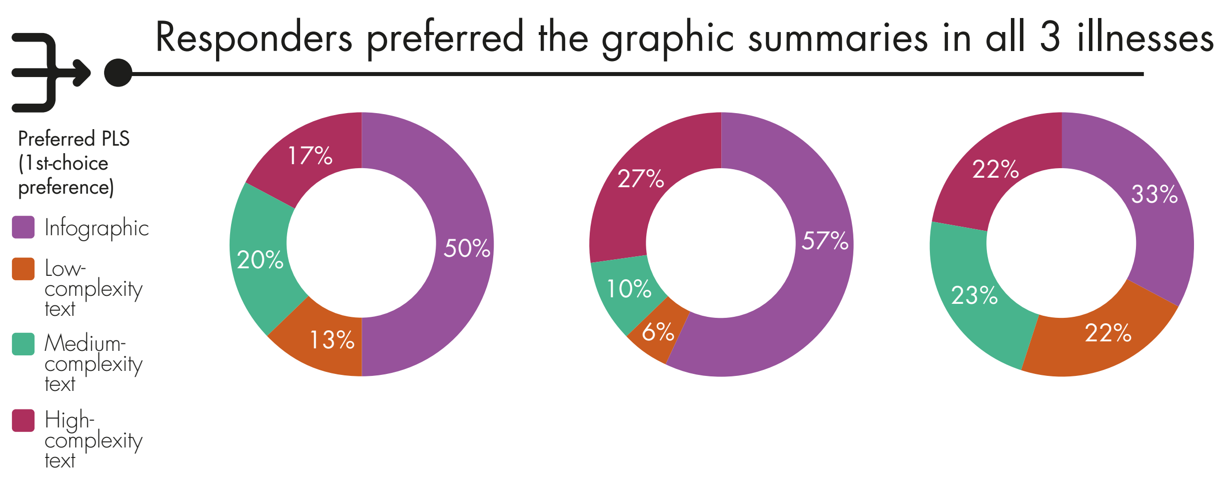

Responders preferred medium-complexity wording (14-17 years' reading age) for text summaries

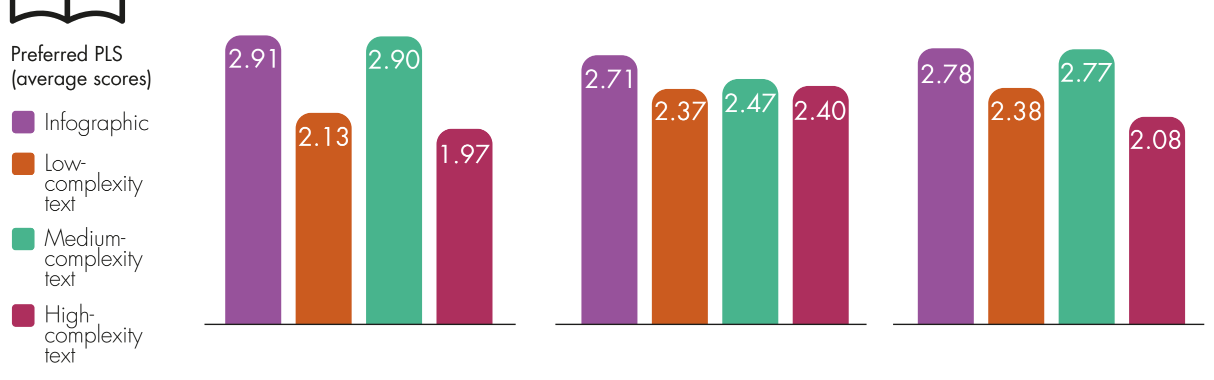

What were the limitations of the study?

More female responders than expected

More people responded to rheumatoid arthritis surveys than psoriasis and multiple sclerosis surveys

What's next?

Authors of original research articles should remember these results when they write summaries for patients

Who sponsored this study?
This research was sponsored by Manchester Metropolitan University and was supported by CMC Connect, McCann Health Medical Communications

The authors would like to thank of all the people who took part in this study

Further information
The full title of this article is: How Can We Optimize the Readability and Format of Plain Language Summaries for Medical Journal Articles? A Cross-Sectional Survey Study
