# Supplementary material for: Optimizing Readability and Format of Plain Language Summaries for Medical Research Articles: Cross-sectional Survey Study
Source: J Med Internet Res. 2022 Jan 11;24(1):e22122. doi: 10.2196/22122 (PMC8790687; doi:10.2196/22122)
Supplement: Multimedia Appendix 2 [file jmir_v24i1e22122_app2.pdf]

PSORIASIS

Warren RB, Mrowietz U, von Kiedrowski R, Niesmann J, Wilsmann-Theis D, Ghoreschi K et al. An intensified dosing schedule of subcutaneous methotrexate in patients with moderate to severe plaque-type psoriasis (METOP): a 52 week, multicentre, randomised, double-blind, placebo-controlled, phase 3 trial. The Lancet. 2017;389(10068):528-537.

HIGH-COMPLEXITY PLAIN LANGUAGE SUMMARY

<u>Subcutaneous methotrexate improves symptoms in patients with moderate-to-severe plaque psoriasis</u>

Researchers have found evidence of the effectiveness of subcutaneous methotrexate (MTX) in patients with moderate-to-severe plaque psoriasis treated for up to 52 weeks. They claim that subcutaneous MTX has a positive risk-benefit profile, and that the route of administration and a more intense dosing schedule should be considered when MTX is used in this patient group.

MTX is a cost-effective therapy that meets the World Health Organization's requirement of having accessible therapies for psoriasis worldwide. Previous research has suggested that patients with failure of oral MTX might benefit from switching to subcutaneous MTX, and a study showed that subcutaneous MTX was more effective than when taken orally in patients with rheumatoid arthritis.

In this study, researchers evaluated the efficacy of subcutaneous MTX in patients with psoriasis. They assigned 120 patients to two groups: 91 to 17.5 mg of subcutaneous MTX once per week, and 29 to subcutaneous placebo injection once per week for 52 weeks. Patients on placebo switched to subcutaneous MTX at Week 16. Patients in the MTX group who did not achieve a 50% improvement in psoriasis symptoms by Week 8 had their dose increased to 22.5 mg MTX once a week. Researchers used the Psoriasis Area and Severity Index (PASI) score to record the redness, thickness and scaling of the skin with psoriasis to measure the improvement in psoriasis symptoms. Achieving a PASI 75 score implies that the patient shows a 75% improvement in psoriasis symptoms.

By Week 52 of the study, 45% of patients had achieved PASI 75 in the methotrexate group compared with 34% in the placebo/MTX group.

The most common adverse events (AEs) during the full study were infections (nasopharyngitis accounted for more than 50% of these AEs) and gastrointestinal disorders. Infections were more common in the placebo/MTX group (50%) than in the MTX group (41%); gastrointestinal disorders were more common in placebo/MTX group (32%) than in the MTX group (13%). Nine patients discontinued the study due to AEs: seven in the MTX group and two in the placebo/MTX group.

Because in this study subcutaneous MTX was compared with placebo, further studies comparing MTX injections to MTX tablets will be required to confirm which is more effective for psoriasis.

MEDIUM-COMPLEXITY PLAIN LANGUAGE SUMMARY

Methotrexate is effective when injected into the skin in patients with psoriasis

Researchers have found that a drug called methotrexate is effective when injected into the skin of patients with psoriasis. They claim that there is a good balance of benefit versus side effects with this treatment. They also think that doctors should consider using methotrexate injections, even at higher doses, when treating patients with psoriasis.

Methotrexate tablets are widely used for treating patients with psoriasis. Previous studies suggest that patients might benefit in switching from methotrexate tablets to injected methotrexate. In another disease, it was found that methotrexate was more effective when injected than when taken orally as a tablet. However, little is known about methotrexate injections in psoriasis.

In this study, researchers divided 120 patients with psoriasis into two groups. One group received a weekly injection of methotrexate. The second group received a weekly injection of placebo and switched to methotrexate in the fourth month. Patients taking methotrexate had a dose increase at the second month if their psoriasis symptoms had not improved by 50%. Patients were treated for up to 1 year. Researchers measured improvement with a score called the PASI. This score measures the redness, thickness and scaling of psoriasis. Achieving a score of PASI 75 means that a patient’s psoriasis improves by 75% or more.

At the end of the study, almost half of the patients had achieved PASI 75 in the methotrexate group compared with a third of patients in the placebo group.

The most common side effects were infections (a common cold in most cases) and gastrointestinal problems. Infections were slightly more common in the placebo group than in the methotrexate group. Gastrointestinal problems were two times more common in the placebo group than in the methotrexate group. Nine patients stopped treatment due to side effects: seven patients in the methotrexate group and two patients in the placebo group.

In this study methotrexate injection was compared with placebo. Next steps would be to compare methotrexate injection with methotrexate tablets to see which is more effective for psoriasis.

LOW-COMPLEXITY PLAIN LANGUAGE SUMMARY

Injections of methotrexate may help to treat psoriasis

This study found that methotrexate injections may help patients with psoriasis. There was a good balance of benefits and side effects. The study also showed that injection of higher doses could work well.

Methotrexate tablets are used a lot to treat psoriasis. Research has shown that changing from tablets to injections may help some patients. But we still don’t know much about methotrexate injections in psoriasis.

Doctors split 120 patients into two groups. One group had a methotrexate injection every week. The other group had a placebo injection every week, and then methotrexate after four months. Doctors counted the number of patients whose psoriasis had improved by three-quarters.

After 1 year, more patients got better in the group taking methotrexate than in the group taking placebo.

Most side effects were infections (mainly a cold) and stomach problems. There were more infections in patients that started with placebo. Stomach problems were also more common in this group. Less than one in ten patients had to stop the study because of side effects.

Doctors think that methotrexate injections could work better than tablets. So they now need to do a study to test this.

METHOTREXATE INJECTIONS FOR PSORIASIS

What did the researchers conclude?

- Methotrexate injections may help patients with psoriasis
- There was a good balance of benefits and side effects
- Injection of higher doses of methotrexate could also work well

Why did the researchers do the study?

- Methotrexate injection has not been well investigated for psoriasis

The study

Duration: 1 year

Who was treated?

- 120 patients
- Aged 18+
- Diagnosed with moderate-to-severe plaque psoriasis

75%

25%

How was the study conducted?

Group 1

Placebo › Methotrexate

29 patients

Weekly Injections

All patients

Month 1: Placebo

Month 2 - 4: Patients improving by 50% or more: Placebo; Patients NOT improving by 50% or more: Higher Dose Placebo

All patients

Month 5 - 6: Switch to Methotrexate

Month 7 - 12: Patients improving by 50% or more: Methotrexate; Patients NOT improving by 50% or more: Higher Dose Methotrexate

Group 2

Methotrexate › Methotrexate

29 patients

Weekly Injections

All patients

Month 1: Methotrexate

Month 2 - 12: Patients improving by 50% or more: Methotrexate; Patients NOT improving by 50% or more: Higher Dose Methotrexate

RESULTS

Proportion of patients improving by 75% or more:

	At Month 4	At Month 12
Group 1 Placebo › Methotrexate	10%	34%
Group 2 Methotrexate › Methotrexate	41%	45%

What were the most common side effects over 12 months?

	Infection	Stomach problems
Group 1 Placebo › Methotrexate	50% of patients	32% of patients
Group 2 Methotrexate › Methotrexate	41% of patients	13% of patients

What's next?

Future studies comparing methotrexate injection to oral dosing to confirm its effectiveness and safety.

Please now click back to the survey which is in another tab of your internet browser
