# Supplementary material for: Optimizing Readability and Format of Plain Language Summaries for Medical Research Articles: Cross-sectional Survey Study
Source: J Med Internet Res. 2022 Jan 11;24(1):e22122. doi: 10.2196/22122 (PMC8790687; doi:10.2196/22122)
Supplement: Multimedia Appendix 3 [file jmir_v24i1e22122_app3.pdf]

MULTIPLE SCLEROSIS

Hauser SL, Bar-Or A, Comi G, Giovannoni G, Hartung H, Hemmer B et al. Ocrelizumab versus Interferon Beta-1a in Relapsing Multiple Sclerosis. New England Journal of Medicine. 2017;376(3):221-234.

HIGH-COMPLEXITY PLAIN LANGUAGE SUMMARY

Ocrelizumab demonstrates better outcomes than interferon beta (IFN-β1a) in patients with relapsing-remitting multiple sclerosis (RRMS)

Researchers found that injection of ocrelizumab, a newly available drug, resulted in lower disease activity and disease progression than injection of IFN-β1a in patients with RRMS over a period of 96 weeks.

Developing new therapies for MS is essential because, despite the number of therapies available, patients often continue to experience clinical symptoms and progression of neurologic disability. Ocrelizumab is a manufactured antibody that depletes B cells, which are a type of white blood cell that play an important role in MS.

Researchers in this report undertook two simultaneous trials (presenting them together) to investigate the efficacy and safety of ocrelizumab when compared with IFN-β1a. Researchers randomly assigned 1656 patients overall to two groups: 825 patients to intravenous infusion of 600 mg of ocrelizumab every 24 weeks, and 826 to subcutaneous injection of 44 μg of IFNβ-1a three times a week, for 96 weeks.

Both studies produced similar results. Researchers found that the rate of relapse was nearly 47% lower in patients receiving ocrelizumab than in patients receiving IFNβ-1a. Other differences in favour of ocrelizumab over IFNβ-1a included a lower proportion of patients with disability progression, a lower number of brain lesions identified with MRI scanning, and a better combined score for walking speed, upper-limb movements and cognition.

The most common adverse events in both groups were infections and infusion/injection-related reactions. Infections were slightly more common with ocrelizumab than with IFN-β1a (59% vs 53%). Infusion-related reactions occurred in 34% of patients treated with ocrelizumab; injection-related reactions occurred in 10% of patients treated with IFN-β1a. Serious infections were lower in patients receiving ocrelizumab (1%) than in patients receiving IFNβ-1a (3%).

Researchers concluded that longer and larger studies would be required to understand more about the safety of ocrelizumab, and to determine whether ocrelizumab offers additional benefits for limiting the progression of disability over the long-term.

MEDIUM-COMPLEXITY PLAIN LANGUAGE SUMMARY

Ocrelizumab is more effective than beta interferon in patients with multiple sclerosis

Researchers have found that a new drug called ocrelizumab was better than beta interferon in a study of patients with relapsing-remitting multiple sclerosis (MS). Ocrelizumab was better at reducing both the activity and the worsening of MS over 2 years.

There are many treatments for MS, but many patients still have relapses and can get worse over time. Ocrelizumab reduces a type of white blood cell that is important in MS.

In this report, researchers ran two identical studies of ocrelizumab versus beta interferon. Both studies were shown together. They divided 1656 patients with MS into two groups. One group received ocrelizumab infused into the vein every 6 months. The second group received beta interferon injected into the skin three times a week.

The results of both studies were similar. Researchers found that patients taking ocrelizumab had nearly half the number of relapses as patients taking beta interferon. Ocrelizumab was also better than beta interferon at slowing the progress of MS. Patients' MRI scans were better with ocrelizumab, as well as walking speed, arm movements and cognition.

Infections and skin reactions related to the injections were common side effects in both groups. There were slightly more injection reactions with ocrelizumab than beta interferon. There were half as many serious infections with ocrelizumab than with beta interferon.

Researchers said that longer and larger studies of ocrelizumab are needed to know more about the side effects and benefits of the drug.

LOW-COMPLEXITY PLAIN LANGUAGE SUMMARY

<u>Ocrelizumab is better than beta interferon in multiple sclerosis</u>

This study found that a new drug called ocrelizumab is better than the beta interferon drug for patients with multiple sclerosis (MS). It was better at reducing the activity of MS. It was also better at stopping MS from getting worse.

There are many drugs for MS. But many patients still have relapses and find that their MS gets worse. Ocrelizumab reduces a type of immune cell that's important in MS. Research has shown that ocrelizumab may improve the symptoms of MS.

Doctors ran two studies that both lasted 2 years. They showed the results of both studies in this report. The doctors split 1656 patients with MS into two groups. One group had ocrelizumab injected into a vein every 6 months. The other group had beta interferon injected into the skin three times a week.

Doctors found that ocrelizumab halved the number of relapses versus beta interferon. It was better at slowing the progress of the MS. It also improved walking, arm movements, and thinking. The brain scans of patients taking ocrelizumab were also better.

The most common side effects with both drugs were infections and problems around where the drug was injected. The injection side effects were slightly more with ocrelizumab. There were half as many serious infections with ocrelizumab than with beta interferon.

Doctors said that longer and larger studies are needed. These will help us to learn more about the side effects and benefits of the drug.

OCRELIZUMAB VERSUS BETA INTERFERON FOR MULTIPLE SCLEROSIS (MS)

What did the researchers conclude?

- Patients on Ocrelizumab, a newly available drug, had less relapses than patients on Beta Interferon
- Patients on Ocrelizumab also had a lower rate of MS progression than patients on Beta Interferon

Why did the researchers do the study?

- There is a need for additional therapies to improve symptoms of MS and slow the progress of MS
- Ocrelizumab is a new drug that reduces a type of white blood cell that's important in MS

The study

Duration: 2 years

Who was treated?

- 1656 patients
- Age 18-55
- Diagnosed with relapsing-remitting MS for over six months

34%

66%

How was the study conducted?

Two identical studies contained 1656 patients in total

1656 patients

Group 1 Ocrelizumab	Group 2 Beta Interferon
827 patients	829 patients
Infusions into the vein	Injections under the skin
Every 6 months	3 times a week

RESULTS AT YEAR 2

For Ocrelizumab versus Beta Interferon:

- **46%** Fewer relapses
- **40%** Lower chance of MS progressing
- **94%** Fewer brain lesions on MRI scanning

Also, better combined score for walking speed, arm movements and cognition

What were the most common side effects over 12 months?

	Side effect		Side effect	
Group 1 Ocrelizumab	Infection	58% of patients	Infusion-related reactions	34% of patients
Group 2 Beta Interferon	Infection	53% of patients	Injection-site redness	16% of patients

What's next?

Longer and larger studies are required in the future to know more about the safety of Ocrelizumab and to see if there are additional benefits for slowing the progress of MS

Please now click back to the survey which is in another tab of your internet browser
