# Supplementary material for: Optimizing Readability and Format of Plain Language Summaries for Medical Research Articles: Cross-sectional Survey Study
Source: J Med Internet Res. 2022 Jan 11;24(1):e22122. doi: 10.2196/22122 (PMC8790687; doi:10.2196/22122)
Supplement: Multimedia Appendix 4 [file jmir_v24i1e22122_app4.pdf]

RHEUMATOID ARTHRITIS

Aletaha D, Bingham C, Tanaka Y, Agarwal P, Kurrasch R, Tak P et al. Efficacy and safety of sirukumab in patients with active rheumatoid arthritis refractory to anti-TNF therapy (SIRROUND-T): a randomised, double-blind, placebo-controlled, parallel-group, multinational, phase 3 study. The Lancet. 2017;389(10075):1206-1217.

HIGH-COMPLEXITY PLAIN LANGUAGE SUMMARY

Sirukumab improves symptoms in difficult-to-treat patients with rheumatoid arthritis

Researchers have found that an investigational drug called sirukumab may be effective in reducing symptoms of rheumatoid arthritis (RA) treated for up to 52 weeks in patients non-responding or intolerant to anti-tumour necrosis factor (TNF) therapy.

Previous research has shown that drugs targeting interleukin-6 (IL-6), a pro-inflammatory molecule in the body, could help improve joint swelling and tenderness, physical function and rate of radiographic progression in patients with RA. In this study, researchers studied sirukumab, a drug that blocks IL-6, in patients with RA that had been unresponsive to at least one anti-TNF therapy.

Researchers assigned 878 patients to one of three groups: 294 to subcutaneous placebo injection every 2 weeks, 292 to 50 mg of subcutaneous sirukumab every 4 weeks, and 292 to 100 mg of subcutaneous sirukumab every 2 weeks. Patients in the placebo group were assigned to either sirukumab dose at Week 24. All patients were at least 18 years of age and had four or more tender joints and four or more swollen joints. Around 60% of patients had previously received two or more biological treatments (including non-TNF drugs), and 81% were taking a disease-modifying antirheumatic drug at the beginning of the study. Researchers used a score called ACR20 to measure the efficacy of sirukumab, representing a 20% improvement in the number of swollen and tender joints and a 20% improvement in other criteria assessing pain, functional ability and markers of inflammation in the blood.

At Week 16, the proportion of patients achieving ACR20 was higher in patients receiving 100 mg or 50 mg sirukumab (45% and 40%) than placebo (24%).

The most common adverse event (AE) throughout the 52 weeks was injection-site redness, which was more common with 100 mg sirukumab (16%) than with 50 mg sirukumab (8%). Thirty-two patients (8%) discontinued the trial in the 50 mg sirukumab group and 52 (12%) in the 100 mg sirukumab group. Serious infections were the most common AE leading to discontinuation of treatment in both groups (4% with 50 mg sirukumab and 5% with 100 mg sirukumab).

Additional studies assessing the safety of sirukumab are being evaluated in a long-term extension study.

MEDIUM-COMPLEXITY PLAIN LANGUAGE SUMMARY

Sirukumab improves joint pain and swelling in patients with rheumatoid arthritis

Researchers have been studying a drug called sirukumab, which is not yet available by prescription. They have found that sirukumab may be effective for patients with rheumatoid arthritis that is difficult to treat.

Interleukin-6 is a chemical in the body that is involved in inflammation. Studies have shown that drugs that block interleukin-6 could help improve rheumatoid arthritis. This study of sirukumab, an interleukin-6 inhibitor, was in patients with rheumatoid arthritis that were not responding to other treatments.

Researchers split 878 patients into three groups and treated them for up to 1 year. One group received placebo twice a month. The second group received a lower dose of sirukumab once a month. The third group received a higher dose of sirukumab twice a month. Patients in the placebo group switched to one of the sirukumab doses after 6 months. All patients were at least 18 and had at least four painful joints and at least four swollen joints. Two-thirds of patients had received two or more other treatments before, and four out of five patients were taking drugs to slow the progress of the disease. Researchers used a scoring system called ACR20 which means 20% improvement in symptoms to work out if the drug was working.

By the fourth month of the study, the number of patients achieving the ACR20 level of improvement was nearly twice as high in patients taking either dose of sirukumab than in patients taking placebo.

The most common side effect by the sixth month was injection-site redness. This was twice as common in patients treated with the higher dose of sirukumab than in patients taking the lower dose. Also, more patients on the higher dose of sirukumab had to stop treatment due to side effects than patients on the lower dose. Infections (mainly pneumonia) were the most common side effect causing patients to stop treatment.

Longer-term studies are being carried out to explore the safety of sirukumab.

LOW-COMPLEXITY PLAIN LANGUAGE SUMMARY

Sirukumab may help to treat rheumatoid arthritis

This study found that a new drug called sirukumab may help to treat patients with rheumatoid arthritis.

There are many drugs to help improve rheumatoid arthritis, but they don't work in every patient. Patients like these are hard to treat.

Research has shown that blocking a chemical in the body called IL-6 could improve rheumatoid arthritis. In this study, doctors used a new drug called sirukumab. They hoped the drug would stop IL-6 from causing swelling and pain.

They found 878 adult patients who had swollen and painful joints and were hard to treat. These patients were split into three groups and were treated for up to 1 year. Group one had a lower dose of sirukumab. Group two had a higher dose of sirukumab. Group three had placebo, and after 6 months these patients started taking the lower or higher dose of sirukumab. All drugs were given through an injection into the skin.

After 4 months of treatment, nearly a quarter of patients on placebo had improved. For the groups that were taking sirukumab, almost half of all the patients improved.

The most common side effect was redness at the injection site. This was more common with the higher dose of sirukumab. Infections like pneumonia were the most common side effect that caused patients to stop their treatment, especially in the group getting the higher dose.

More studies are being carried out to learn more about the long-term safety of sirukumab. Sirukumab is not yet available from doctors.

SIRUKUMAB INJECTIONS FOR RHEUMATOID ARTHRITIS

What did the researchers conclude?

Sirukumab is effective in reducing the symptoms of rheumatoid arthritis in a difficult-to-treat population over one year

Why did the researchers do the study?

- To investigate if blocking a chemical in the body called IL-6 that causes inflammation could help improve rheumatoid arthritis
- Researchers tested a drug called Sirukumab to block IL-6

The study

Duration: 1 year

Who was treated?

- 878 patients
- Aged 18+
- Diagnosed with rheumatoid arthritis
- Not responding to, or not tolerating, other therapies

19%

81%

How was the study conducted?

878 patients split into three groups

878 patients

Group 1	Group 2	Group 3
Placebo	Lower Dose Sirukumab	Higher Dose Sirukumab

Group 1

Placebo

294 patients

Injections every 2 weeks

Month 1-6: All patients – Placebo

Month 6-12: Half of the placebo patients – Lower Dose Sirukumab; Half of the placebo patients – Higher Dose Sirukumab

Group 2

Lower Dose Sirukumab

292 patients

Injections every 4 weeks

Month 1-12: All patients – Lower Dose Sirukumab

Group 3

Higher dose Sirukumab

292 patients

Injections every 2 weeks

Month 1-12: All patients – Higher Dose Sirukumab

RESULTS:

Patients improving by 20% or more at Month 4

Group 1	Group 2	Group 3
Placebo	Lower Dose Sirukumab	Higher Dose Sirukumab
24%	40%	45%

What were the most common side effects over 12 months?

	Injection-site redness		Serious Infection	
Group 1 Placebo	8%	of patients	4%	of patients*
Group 2 Lower Dose Sirukumab	16%	of patients	5%	of patients*

*Pneumonia in 1% of patients with serious infections

What's next?

Additional studies are being carried out to know more about the safety of Sirukumab over a longer period of time

Please now click back to the survey which is in another tab of your internet browser
