# Supplementary material for: Optimizing Readability and Format of Plain Language Summaries for Medical Research Articles: Cross-sectional Survey Study
Source: J Med Internet Res. 2022 Jan 11;24(1):e22122. doi: 10.2196/22122 (PMC8790687; doi:10.2196/22122)
Supplement: Multimedia Appendix 5 [file jmir_v24i1e22122_app5.pdf]

PSORIASIS TEXT: READING AGE AND OTHER FEATURES

	LOW-COMPLEXITY TEXT PLS	MEDIUM-COMPLEXITY TEXT PLS	HIGH-COMPLEXITY TEXT PLS	ORIGINAL ABSTRACT*
READING AGE	12-14 years old (US Grade 7-8)	15-17 years old (US Grade 10-11)	18-19 years old (US Grade 12)	University / college graduate
WORD COUNT	193	332	371	413
NUMBER OF SENTENCES	17	22	17	17
AVG. WORDS PER SENTENCE	11	15	22	24
POLYSYLLABIC WORDS (3+ SYLLABLES)	24 (12%)	61 (18%)	71 (19%)	94 (23%)
ACRONYMS	None	PASI	MTX, AE, PASI	METOP, PASI, CI

*Text abridged to remove data in parentheses that were disrupting the automatic readability assessment
Abstract body text (excluding funding); assessed using http://www.readabilityformulas.com/free-readability-formula-tests.php

MULTIPLE SCLEROSIS TEXT: READING AGE AND OTHER FEATURES

	LOW-COMPLEXITY TEXT PLS	MEDIUM-COMPLEXITY TEXT PLS	HIGH-COMPLEXITY TEXT PLS	ORIGINAL ABSTRACT*
READING AGE	11-13 years old (US Grade 6-7)	15-17 years old (US Grade 10-11)	University / college graduate	17-18 years old (US Grade 12)
WORD COUNT	241	240	317	342
NUMBER OF SENTENCES	21	17	13	22
AVG. WORDS PER SENTENCE	11	14	24	16
POLYSYLLABIC WORDS (3+ SYLLABLES)	28 (12%)	46 (19%)	83 (26%)	82 (24%)
ACRONYMS	MS	MS, MRI	IFNβ-1a, RRMS, MS, MRI	CD20+, CI

*Text abridged to remove data in parentheses that were disrupting the automatic readability assessment
Abstract body text (excluding funding); assessed using http://www.readabilityformulas.com/free-readability-formula-tests.php

RHEUMATOID ARTHRITIS TEXT: READING AGE AND OTHER FEATURES

	LOW-COMPLEXITY TEXT PLS	MEDIUM-COMPLEXITY TEXT PLS	HIGH-COMPLEXITY TEXT PLS	ORIGINAL ABSTRACT*
READING AGE	12-14 years old (US Grade 7-8)	14-15 years old (US Grade 9)	University / college graduate	University / college graduate
WORD COUNT	252	328	367	670
NUMBER OF SENTENCES	19	19	13	22
AVG. WORDS PER SENTENCE	13	17	28	30
POLYSYLLABIC WORDS (3+ SYLLABLES)	25 (10%)	41 (13%)	73 (20%)	145 (22%)
ACRONYMS	IL-6	ACR20	RA, TNF, IL-6, ACR20, AE	TNF, DMARD, ACR20, CI

*Text abridged to remove data in parentheses that were disrupting the automatic readability assessment
Abstract body text (excluding funding); assessed using http://www.readabilityformulas.com/free-readability-formula-tests.php
