# Supplementary material for: Optimizing Readability and Format of Plain Language Summaries for Medical Research Articles: Cross-sectional Survey Study
Source: J Med Internet Res. 2022 Jan 11;24(1):e22122. doi: 10.2196/22122 (PMC8790687; doi:10.2196/22122)
Supplement: Multimedia Appendix 6 [file jmir_v24i1e22122_app6.pdf]

MMU Publication Lay Summary research 2018

Survey

Survey on [Psoriasis / Multiple Sclerosis / Rheumatoid Arthritis]: How can we improve communication of medical research to patients and carers?

Hi! My name is Leia and I'm a student at the Manchester Metropolitan University. I'm studying for an MSc in Science Communication. As part of this, I'm undertaking a survey to understand how we can improve communication of medical research to patients and carers.

Based on a recent scientific publication in an important medical journal, I've written 3 text summaries of different complexity and 1 graphic summary.

I'd be really grateful for your help in reading these summaries and completing a survey to obtain your feedback on each one. At the end of the survey, I'm also asking for your overall preferences. I anticipate that the whole process should take approximately 20-30 minutes. Your responses are anonymous and your feedback will be kept confidential. The results of this survey will be used for scholarly purposes – hopefully to improve how we communicate medical information to patients and carers.

Your participation in this research study is voluntary and very much appreciated. If you do have any questions with regards to this survey, please feel free to contact me at leia.m.martinez-silvagnoli@stu.mmu.ac.uk.

Thank you!

Leia Martínez Silvagnoli

1 With which gender do you identify?

Male

Female

Other

2 Please select your age group

18-24

25-34

35-44

45-54

55-64

65+

3 What is the highest level of education that you have achieved?

GCSE

A-Level

Foundation degree (e.g. diploma)

Bachelor's degree

Masters/ PhD /other post- graduate

Other (please specify)

Please click on this text to read the first text summary

4 How easy/difficult did you find it to read the summary?

Easy to read

Somewhat easy to read

Difficult to read

Very difficult to read

5 What do you think about the length of the summary?

Too short

About the right length

Too long

6 Is there enough detail in the summary to understand the study?

Not enough detail

Yes, about the right amount of detail

Too much detail

Please click on this text to read the second text summary (same topic; different writing style)

7 How easy/difficult did you find it to read the summary?

Easy to read

Somewhat easy to read

Difficult to read

Very difficult to read

8 What do you think about the length of the summary?

Too short

About the right length

Too long

9 Is there enough detail in the summary to understand the study?

Not enough detail

Yes, about the right amount of detail

Too much detail

Please click on this text to read the third text summary (same topic; different writing style)

10 How easy/difficult did you find it to read the summary?

Easy to read

Somewhat easy to read

Difficult to read

Very difficult to read

11 What do you think about the length of the summary?

Too short

About the right length

Too long

12 Is there enough detail in the summary to understand the study?

Not enough detail

Yes, about the right amount of detail

Too much detail

Please click on this text to read a graphic summary (same topic; different style)

13 How easy/difficult did you find it to read the summary?

Easy to read

Somewhat easy to read

Difficult to read

Very difficult to read

14 What do you think about the length of the summary?

Too short

About the right length

Too long

15 Is there enough detail in the summary to understand the study?

Not enough detail

Yes, about the right amount of detail

Too much detail

16 Do you think that there is an adequate balance of graphics and text?

Yes

No

Not sure

17 Do you think that publication summaries such as the ones presented in this survey are valuable in keeping you informed about recent research?

Definitely

Probably

Not sure

Probably not

Definitely not

18 Would you feel confident discussing content related to the summaries with a doctor or nurse?

Definitely

Probably

Not sure

Probably not

Definitely not

19 Could you please rank the publication summaries and infographic in order of preference?

1 2 3 4

First text summary

1 2 3 4

Second text summary

1 2 3 4

Third text summary

1 2 3 4

Graphic summary

20 Could you please give the main reasons why you have ranked the summaries in that order?
