# Supplementary material for: Optimizing Readability and Format of Plain Language Summaries for Medical Research Articles: Cross-sectional Survey Study
Source: J Med Internet Res. 2022 Jan 11;24(1):e22122. doi: 10.2196/22122 (PMC8790687; doi:10.2196/22122)
Supplement: Multimedia Appendix 7 [file jmir_v24i1e22122_app7.pdf]

UK-BASED PATIENT ASSOCIATIONS AND FACEBOOK PATIENT GROUPS SURVEYED

Psoriasis

- Psoriasis and Psoriatic Arthritis Alliance (PAPAA)
- Psoriasis Association
- Psoriasis Support Group UK

Multiple Sclerosis

- MS Society
- Multiple Sclerosis Trust
- MS-UK
- Asian MS (UK national support group)
- Multiple Sclerosis Support/Chat Group UK
- Mutual Support (Armed Forces)

Rheumatoid Arthritis

- National Rheumatoid Arthritis Society Regional groups
- Arthritis Research UK
- Arthritis Care (Part of Arthritis Research UK)
- Arthritis Action
- UK Rheumatoid Arthritis Wonky Group
