# Supplementary material for: Optimizing Readability and Format of Plain Language Summaries for Medical Research Articles: Cross-sectional Survey Study
Source: J Med Internet Res. 2022 Jan 11;24(1):e22122. doi: 10.2196/22122 (PMC8790687; doi:10.2196/22122)

PLAIN LANGUAGE SUMMARY PROJECT

CORE AND SUBGROUP ANALYSES

POPULATION PROFILES

- 167 responders in total
  - 1:1:3 for Psoriasis:MS:RA
- Ages of responders as expected for each indication
- Majority were female
- ~50% educated to higher-education degree level

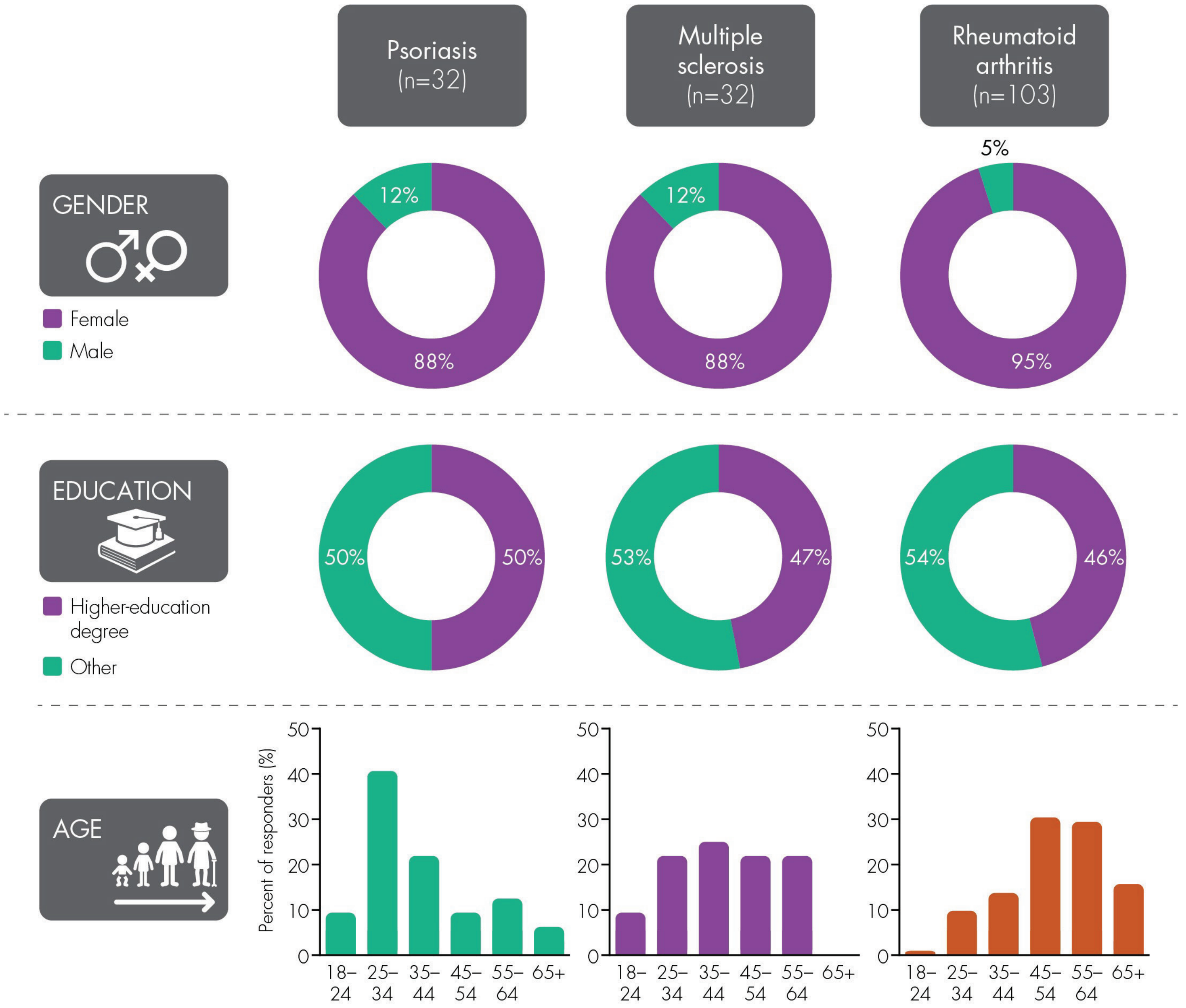

PREFERRED PLS

- **First-choice preference:** Infographic PLS was most popular for all three indications

- **Weighted average preference scores:** Infographic and medium-complexity text PLS (reading age: 14–17 years) ranked highest for all three indications

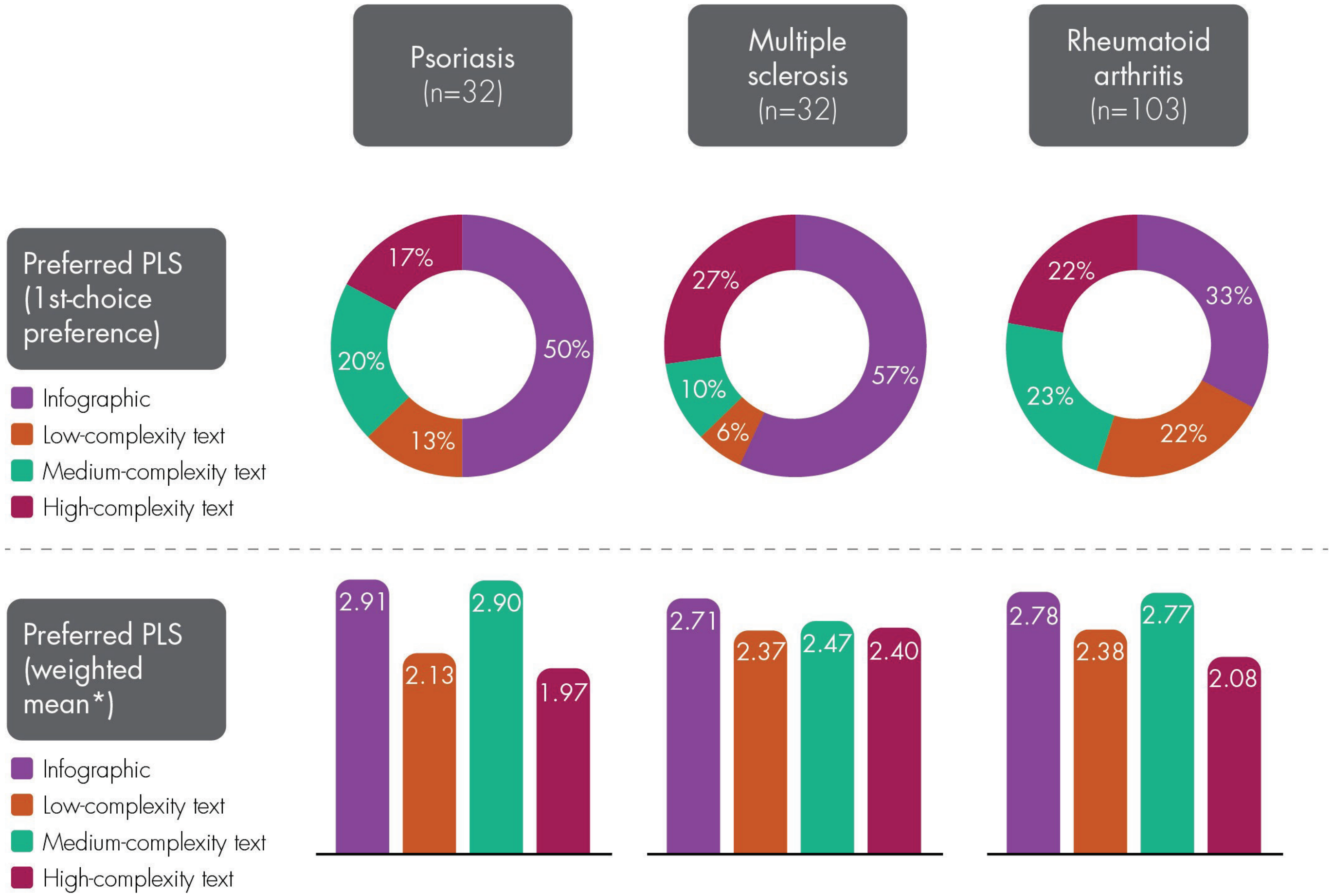

*Weights are applied in reverse: 1st choice has a weight of 4 → 4th choice has a weight of 1

DEMOGRAPHY FOR INFOGRAPHIC SUMMARY PREFERENCE

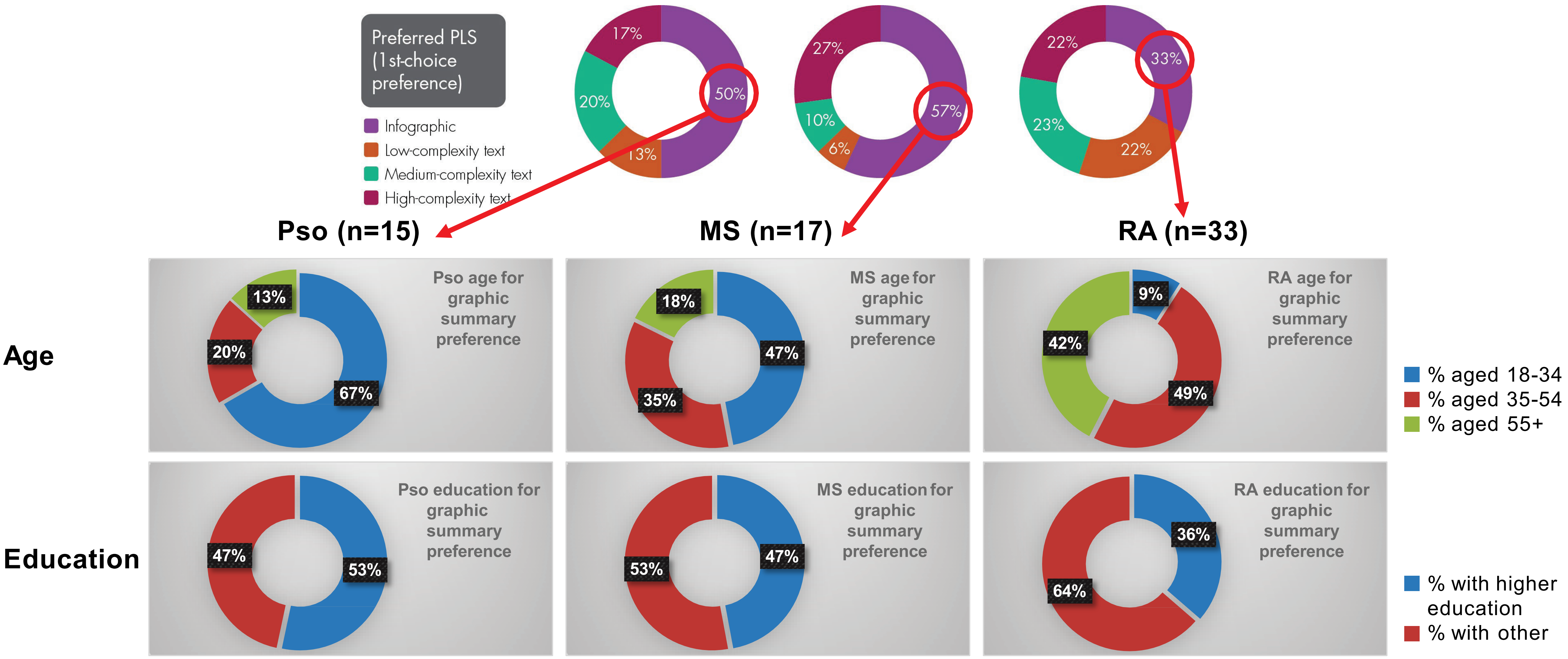

DEMOGRAPHY FOR HIGH-COMPLEXITY TEXT SUMMARY PREFERENCE

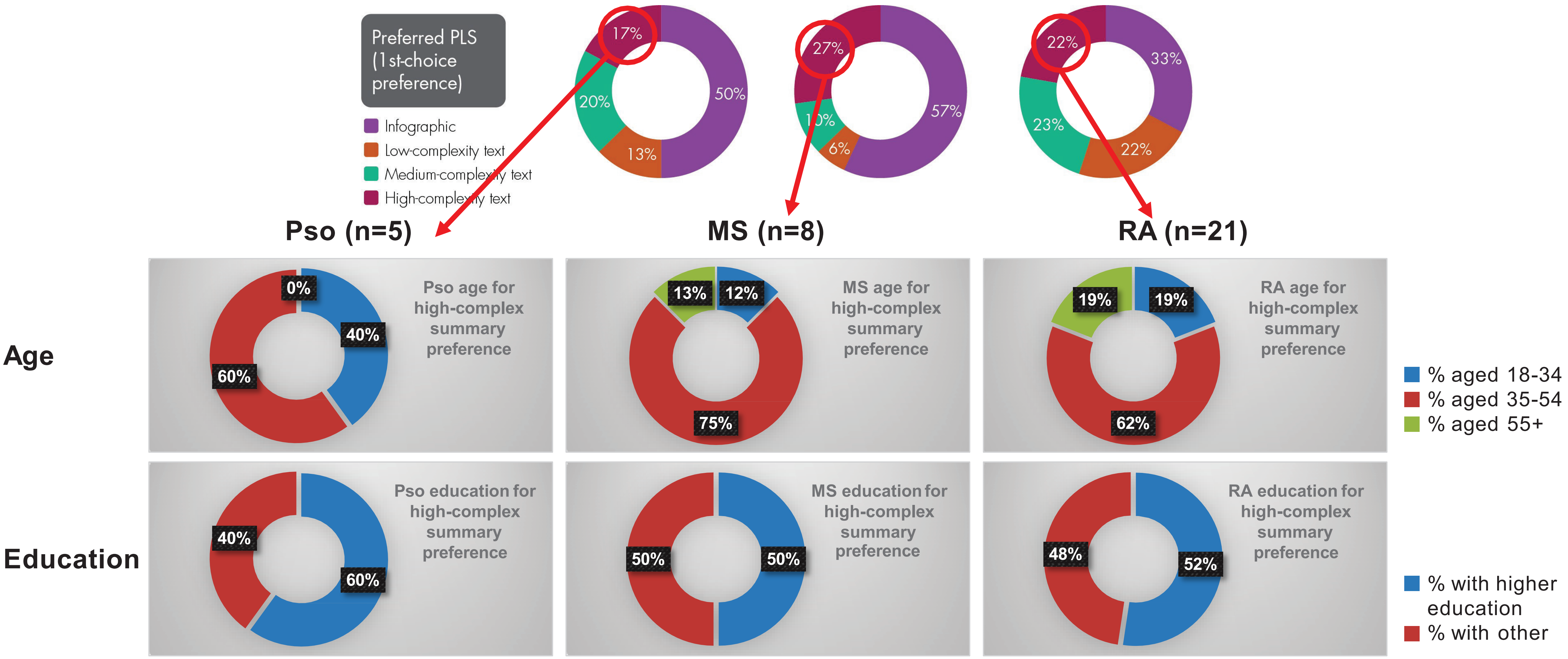

DEMOGRAPHY FOR MEDIUM-COMPLEXITY TEXT SUMMARY PREFERENCE

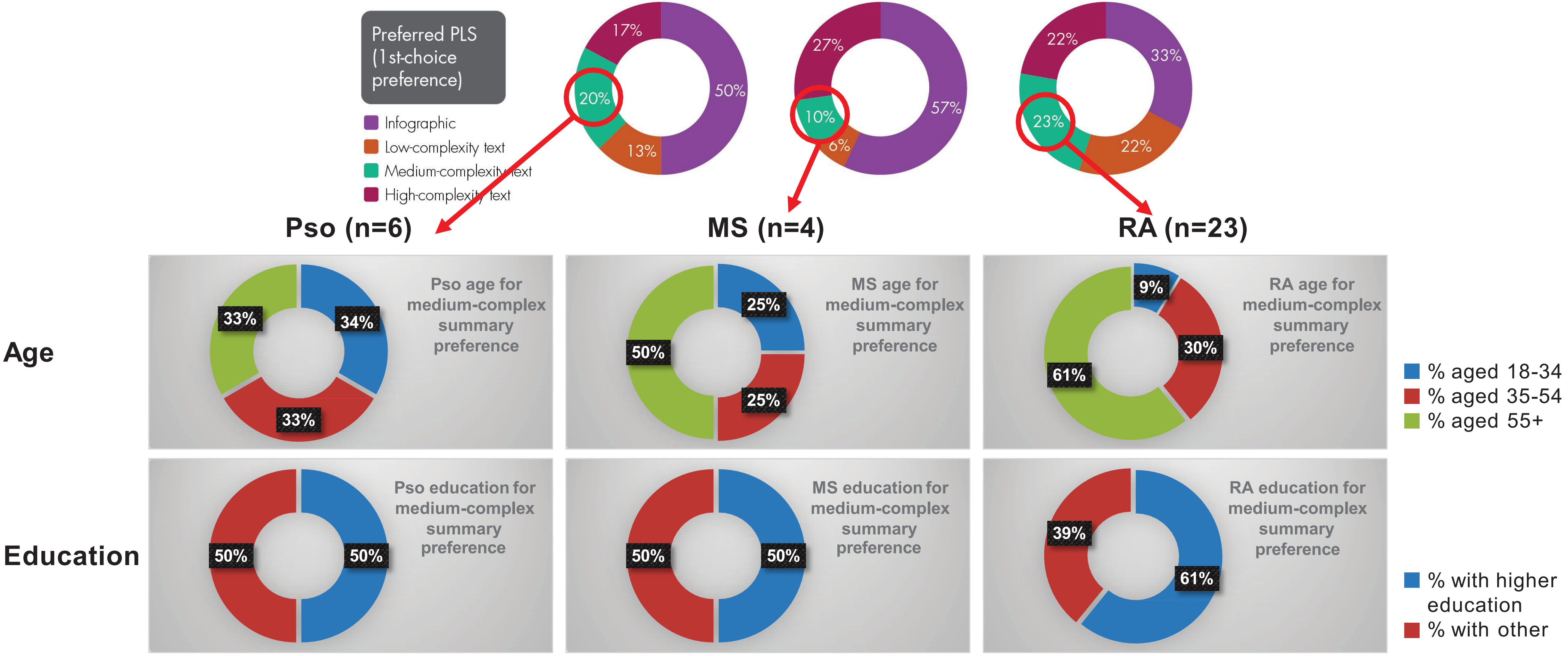

DEMOGRAPHY FOR LOW-COMPLEXITY TEXT SUMMARY PREFERENCE

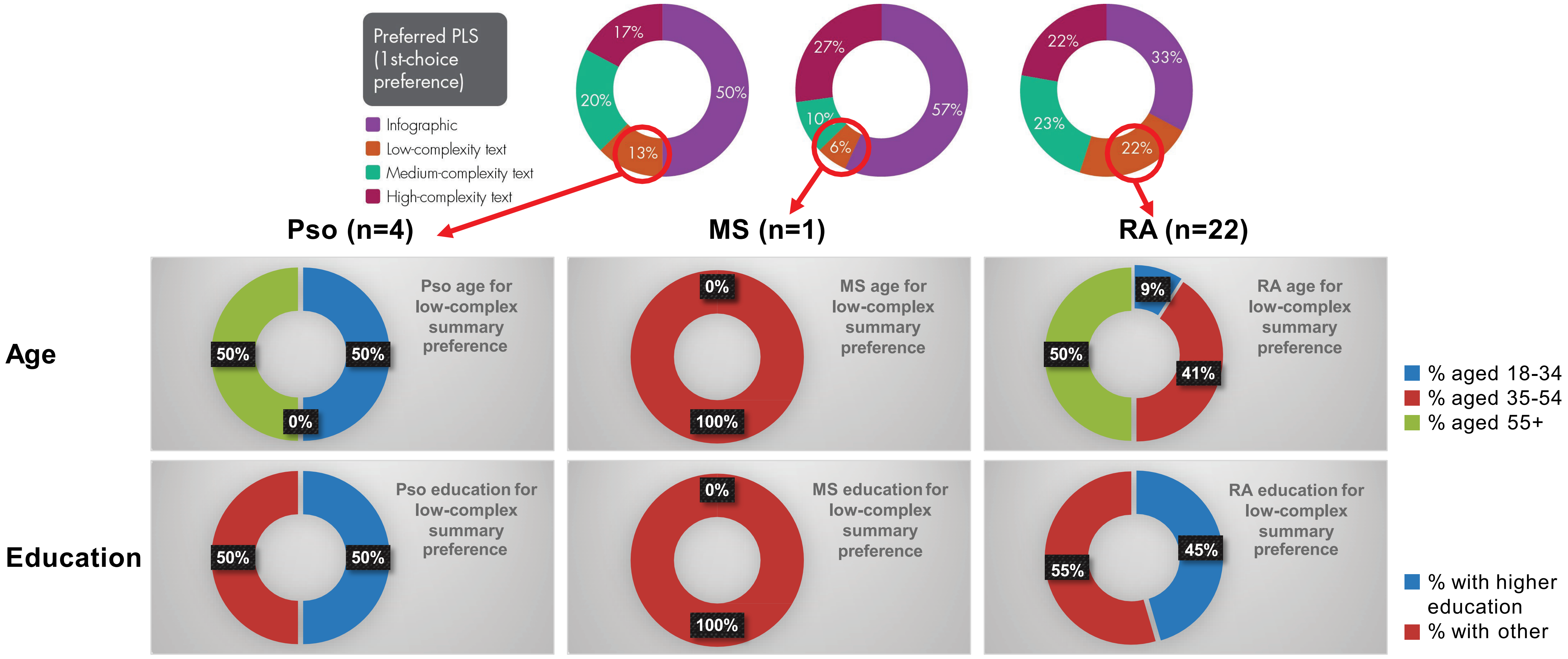

PSORIASIS BY GENDER

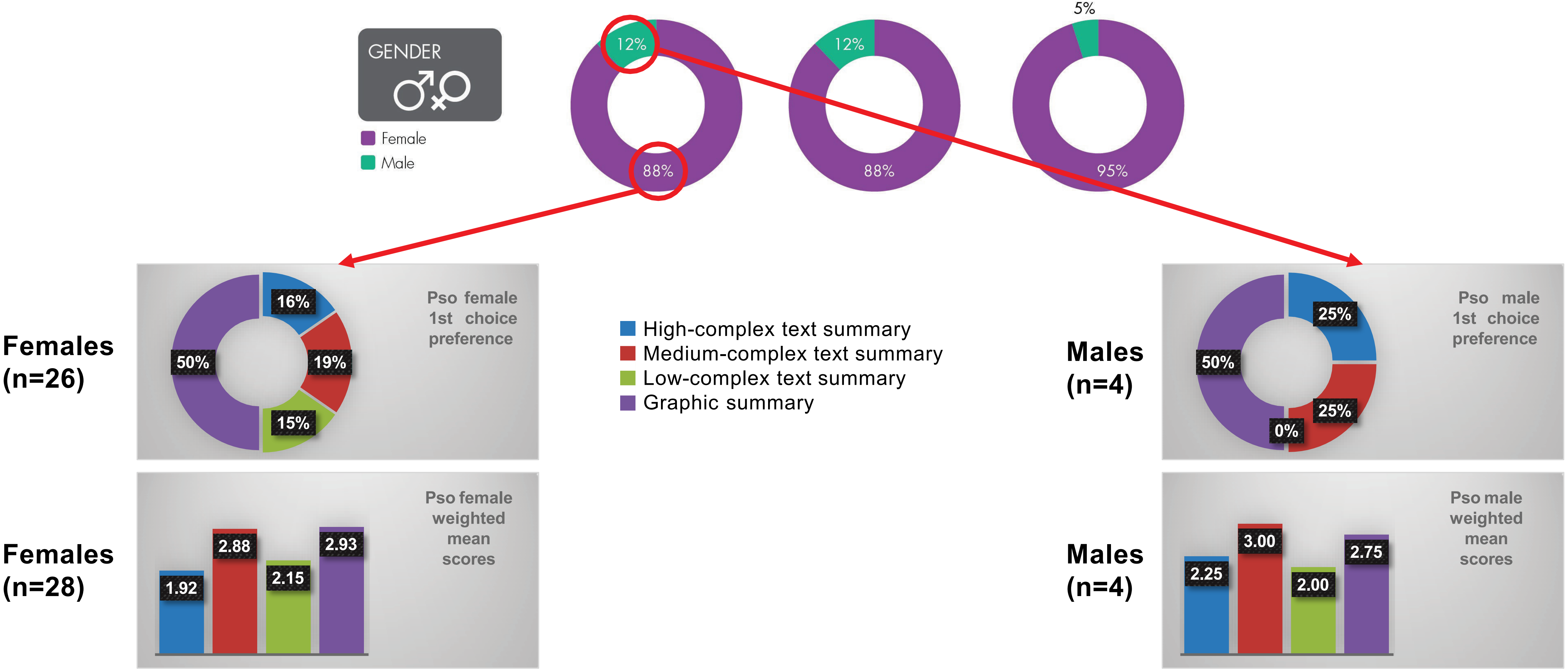

PSORIASIS BY AGE

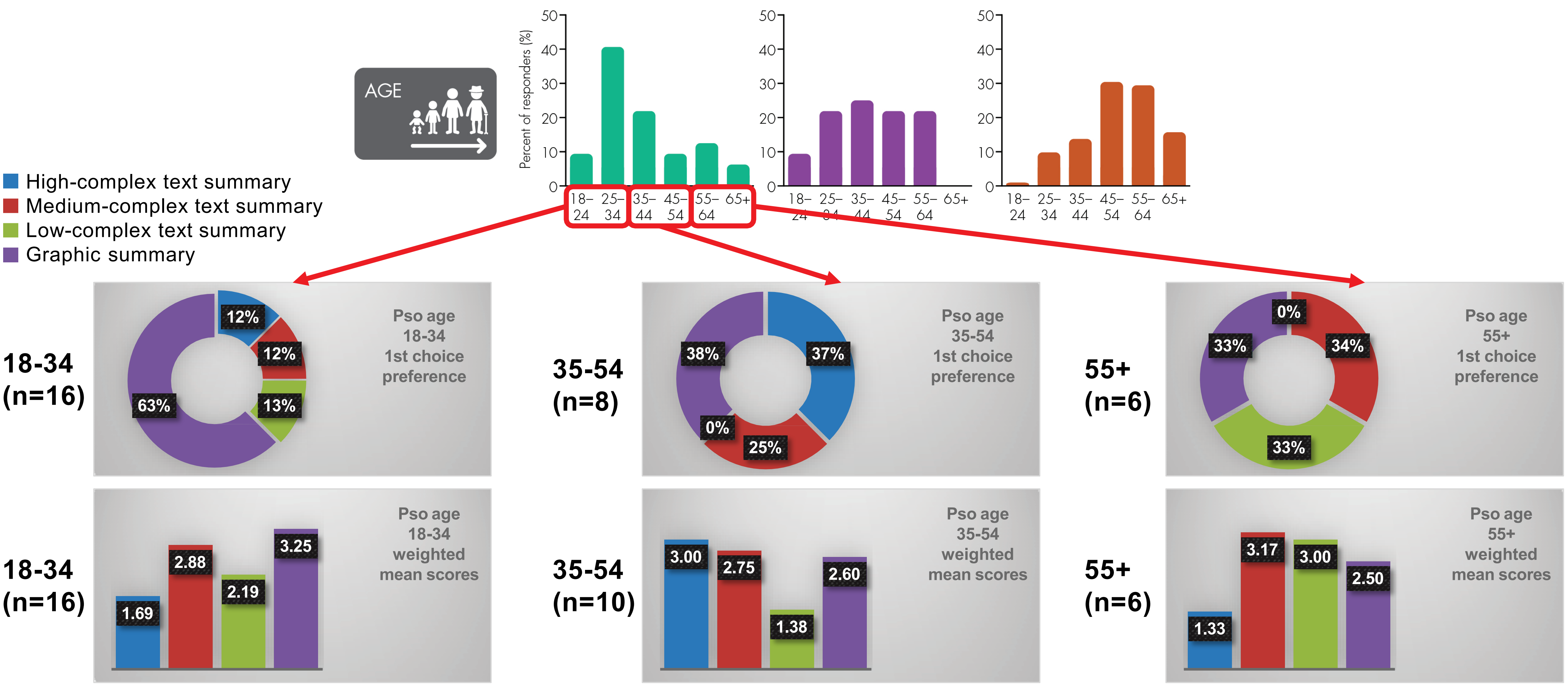

PSORIASIS BY EDUCATION

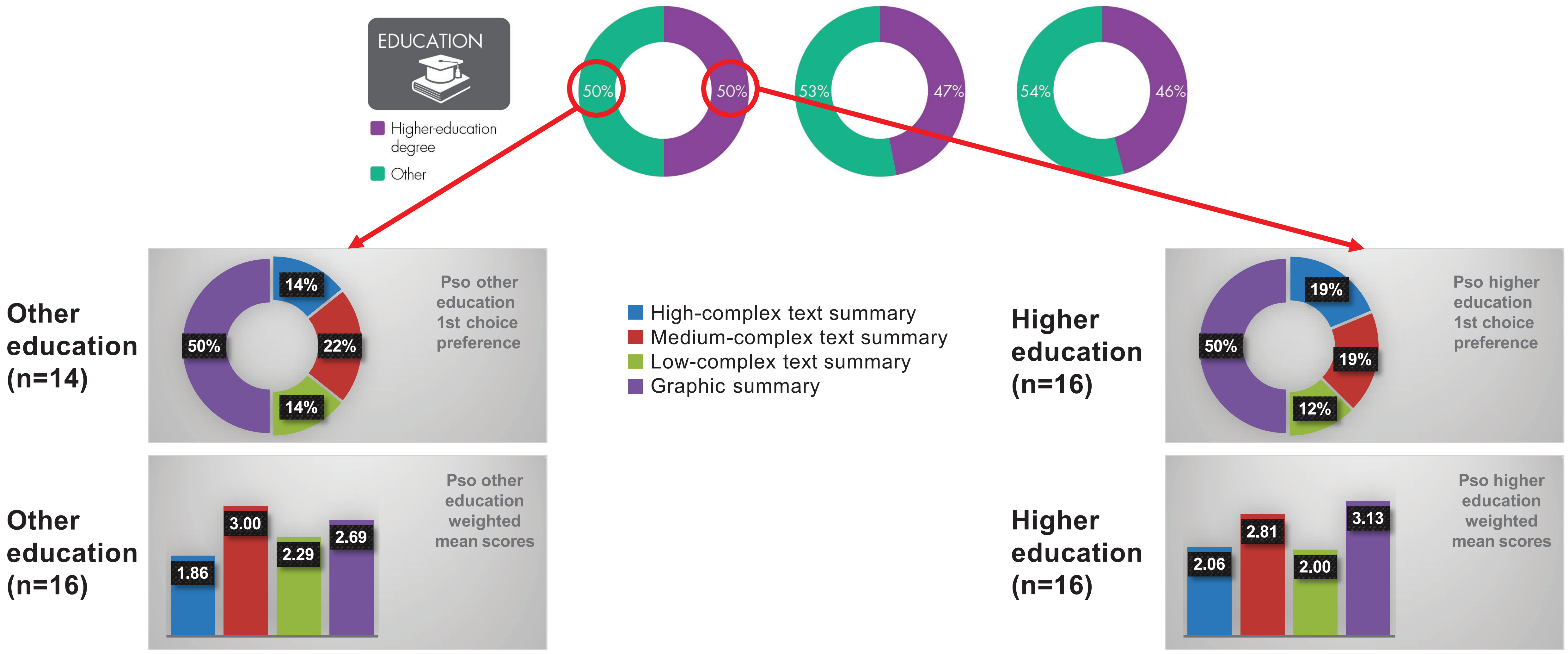

MS BY GENDER

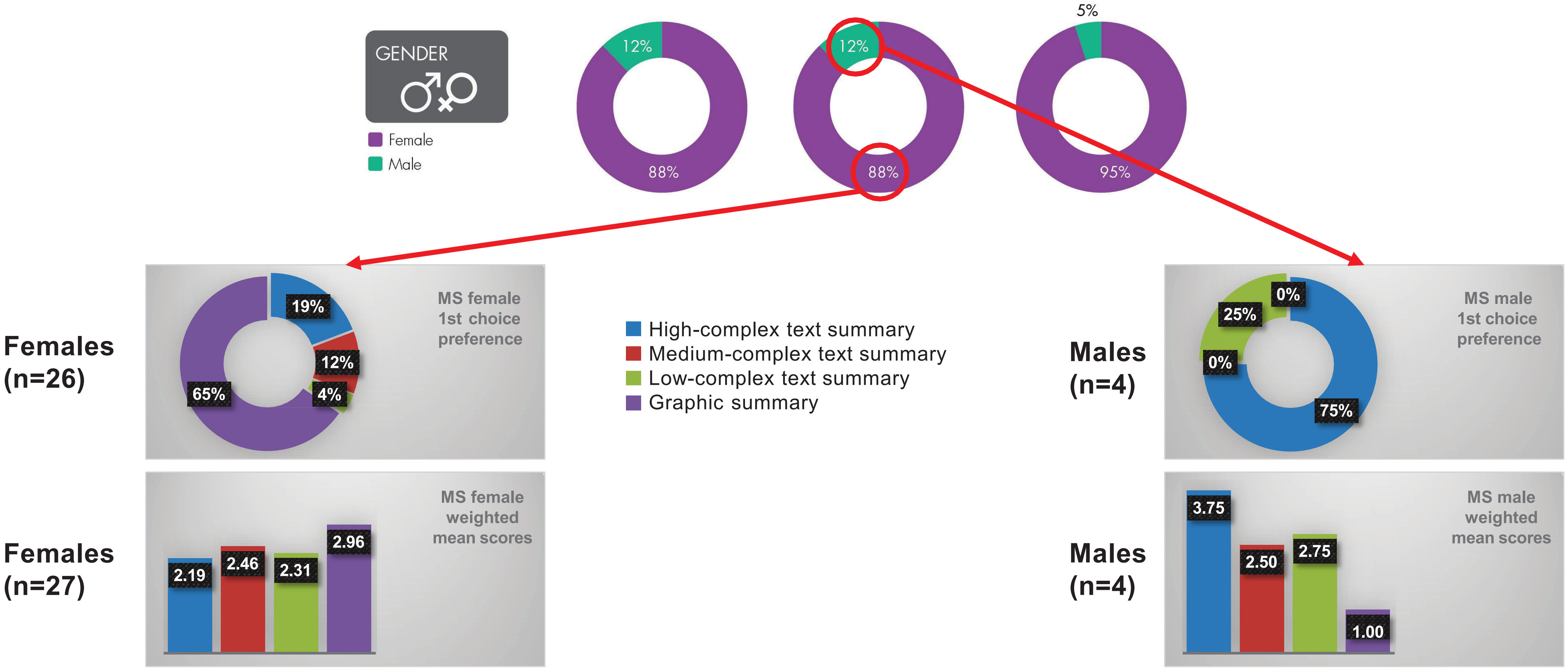
GENDER
Female
Male
12%
88%
12%
88%
5%
95%
Females (n=26)
19%
12%
4%
65%
MS female 1st choice preference
High-complex text summary
Medium-complex text summary
Low-complex text summary
Graphic summary
Males (n=4)
0%
25%
0%
75%
MS male 1st choice preference
Females (n=27)
2.19
2.46
2.31
2.96
MS female weighted mean scores
Males (n=4)
3.75
2.50
2.75
1.00
MS male weighted mean scores

MS BY AGE

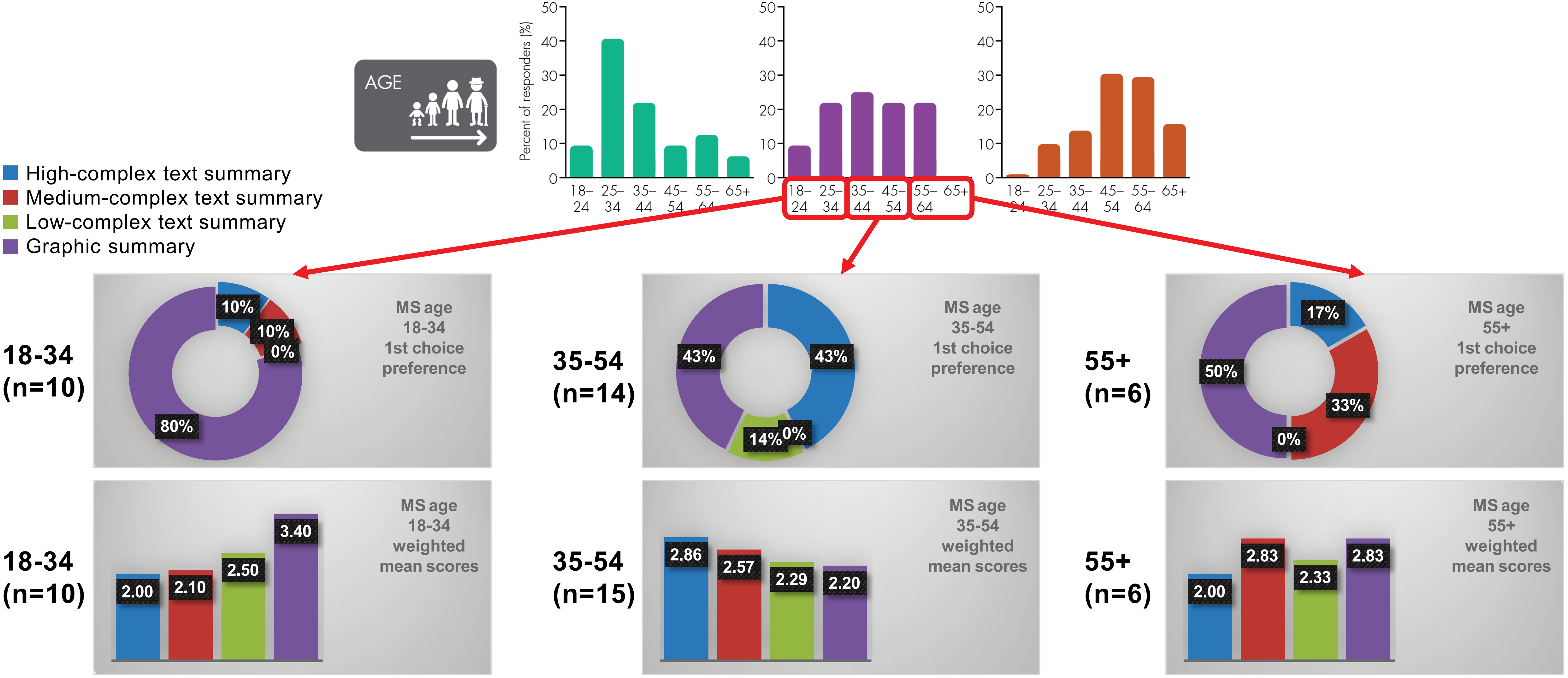
AGE
Percent of responders (%)
50
40
30
20
10
0
18–24
25–34
35–44
45–54
55–64
65+
High-complex text summary
Medium-complex text summary
Low-complex text summary
Graphic summary
18-34 (n=10)
10%
10%
0%
80%
MS age 18-34 1st choice preference
35-54 (n=14)
43%
43%
14%
0%
MS age 35-54 1st choice preference
55+ (n=6)
17%
50%
33%
0%
MS age 55+ 1st choice preference
18-34 (n=10)
2.00
2.10
2.50
3.40
MS age 18-34 weighted mean scores
35-54 (n=15)
2.86
2.57
2.29
2.20
MS age 35-54 weighted mean scores
55+ (n=6)
2.00
2.83
2.33
2.83
MS age 55+ weighted mean scores

MS BY EDUCATION

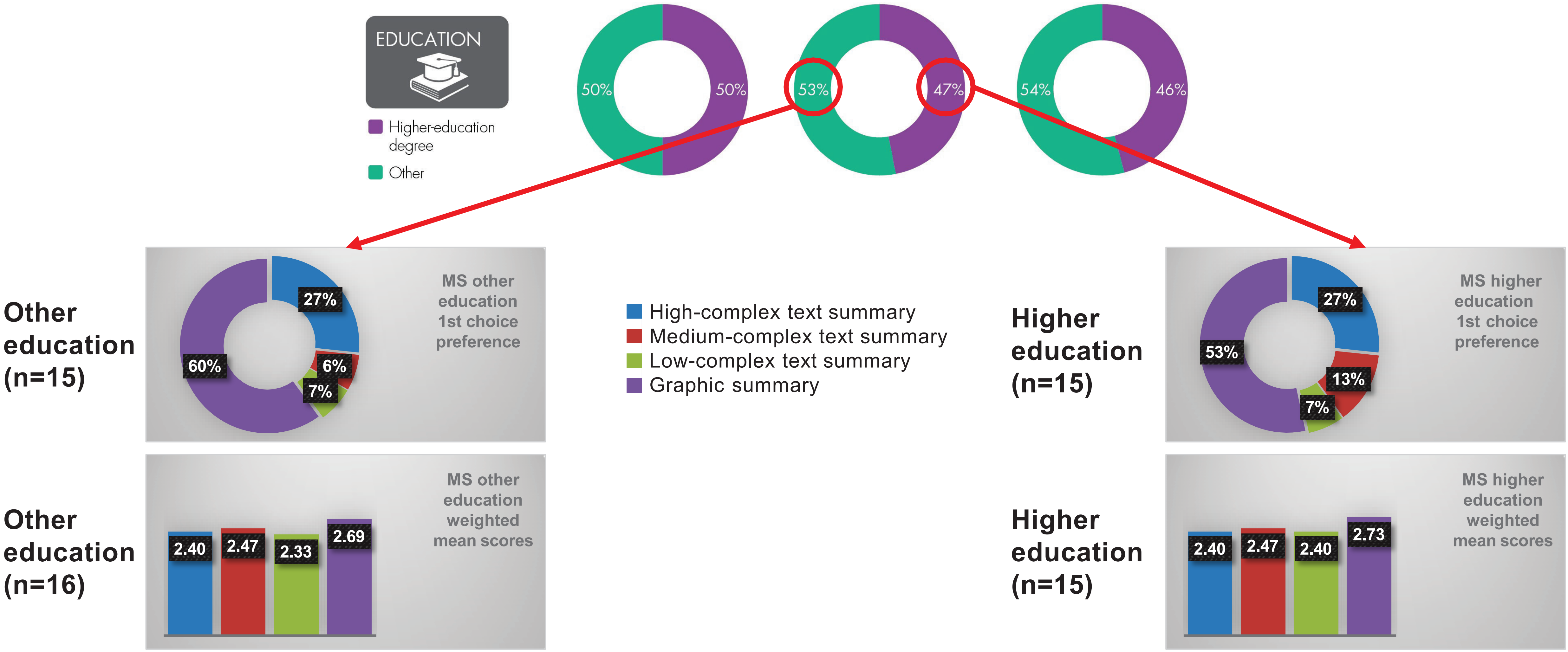

RA BY GENDER

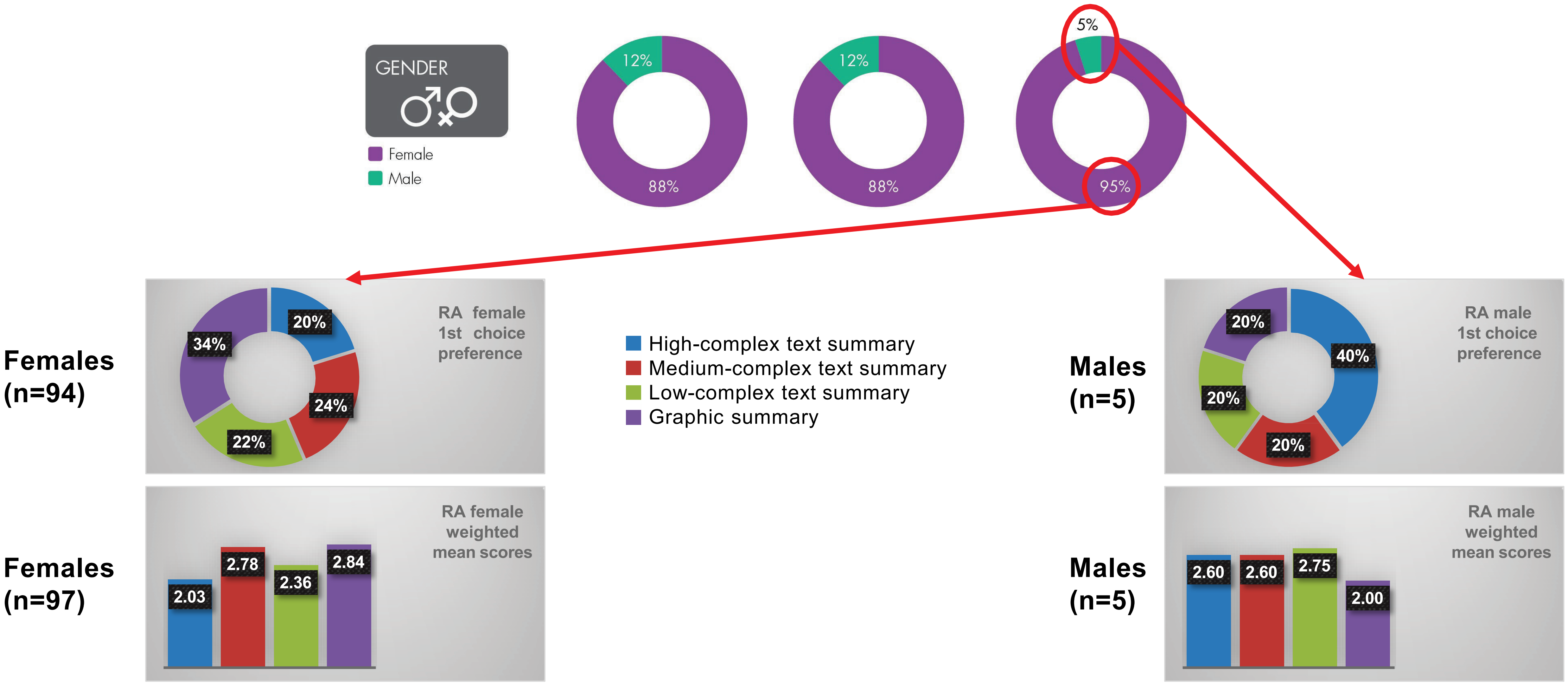
GENDER
Female
Male
12%
88%
12%
88%
5%
95%
Females (n=94)
20%
24%
22%
34%
RA female 1st choice preference
High-complex text summary
Medium-complex text summary
Low-complex text summary
Graphic summary
Males (n=5)
40%
20%
20%
20%
RA male 1st choice preference
Females (n=97)
2.03
2.78
2.36
2.84
RA female weighted mean scores
Males (n=5)
2.60
2.60
2.75
2.00
RA male weighted mean scores

RA BY AGE

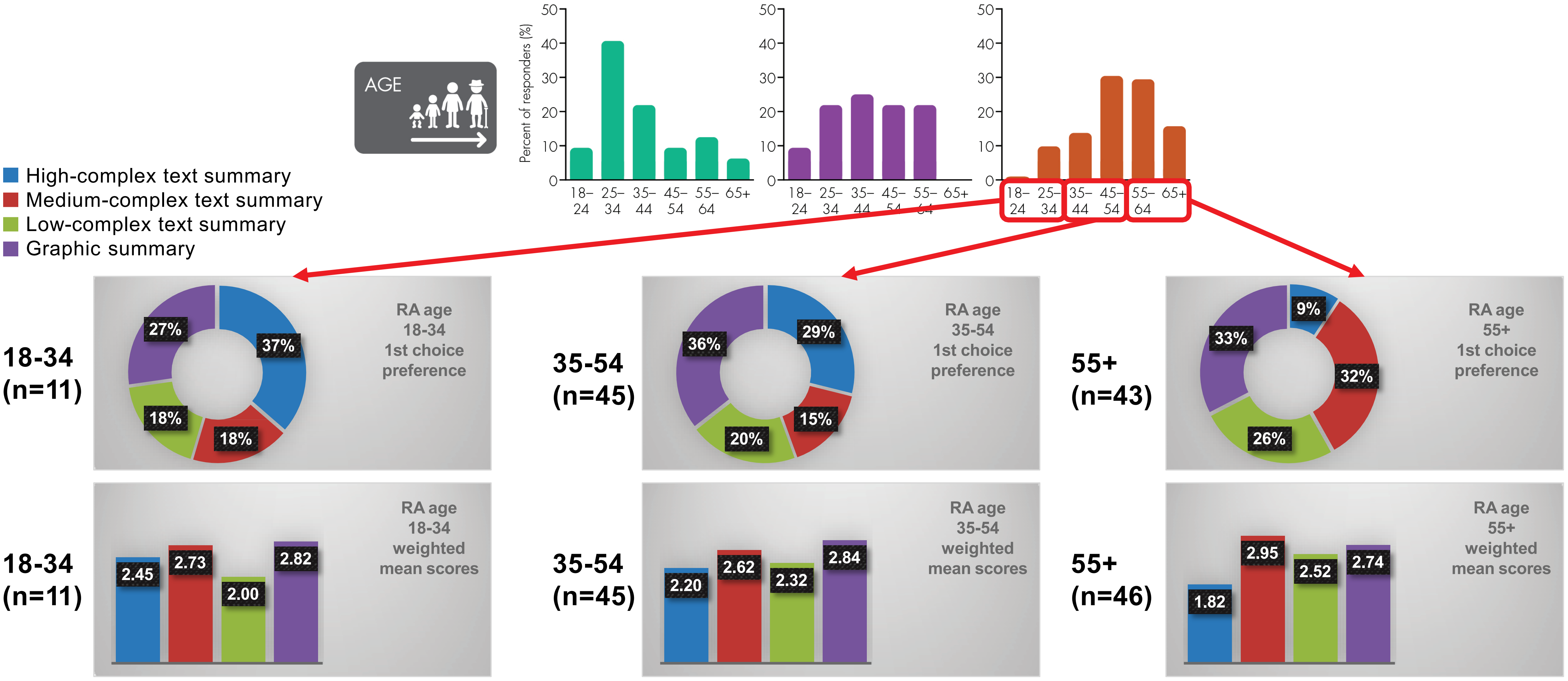

AGE
Percent of responders (%)
50
40
30
20
10
0
18–24
25–34
35–44
45–54
55–64
65+
High-complex text summary
Medium-complex text summary
Low-complex text summary
Graphic summary
18-34 (n=11)
37%
18%
18%
27%
RA age 18-34 1st choice preference
35-54 (n=45)
29%
15%
20%
36%
RA age 35-54 1st choice preference
55+ (n=43)
9%
32%
26%
33%
RA age 55+ 1st choice preference
18-34 (n=11)
2.45
2.73
2.00
2.82
RA age 18-34 weighted mean scores
35-54 (n=45)
2.20
2.62
2.32
2.84
RA age 35-54 weighted mean scores
55+ (n=46)
1.82
2.95
2.52
2.74
RA age 55+ weighted mean scores

RA BY EDUCATION

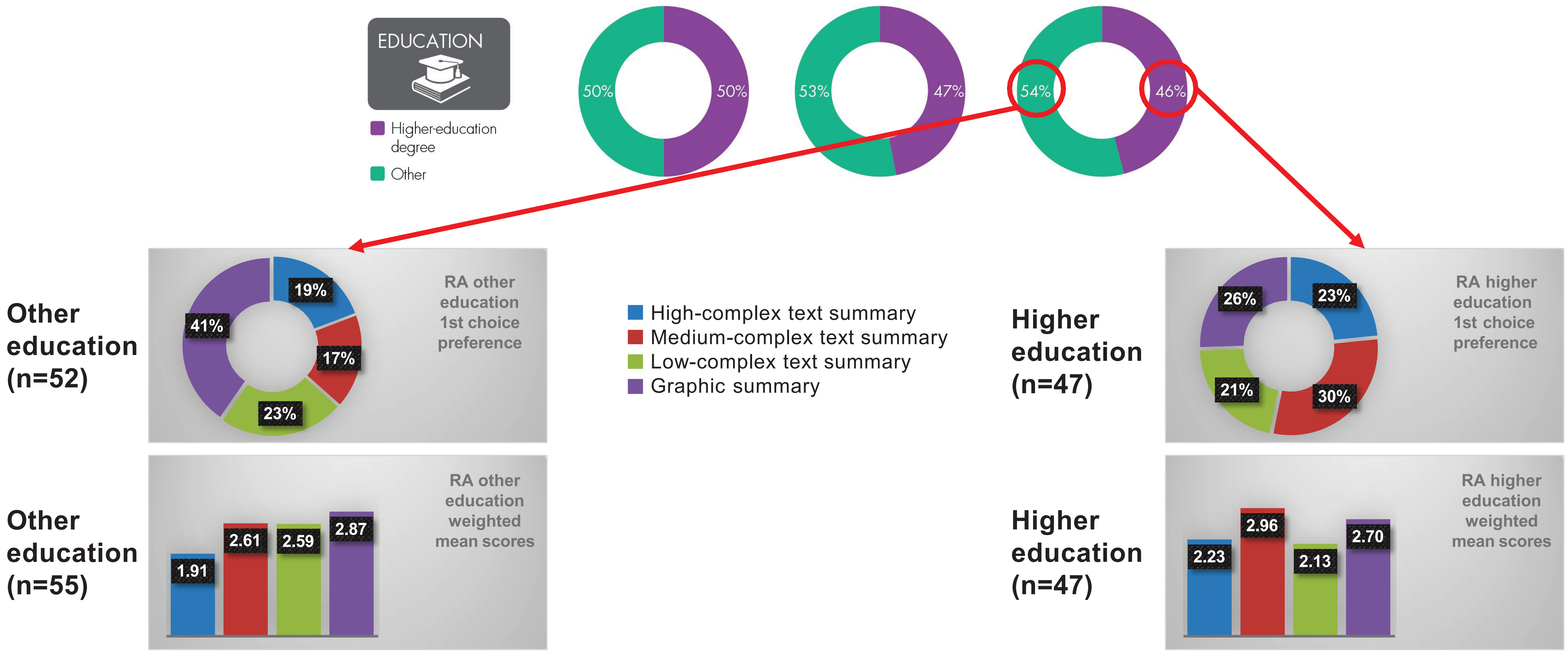

Supplement: Multimedia Appendix 8 [file jmir_v24i1e22122_app8.pdf]
