# Supplementary material for: Optimizing Readability and Format of Plain Language Summaries for Medical Research Articles: Cross-sectional Survey Study
Source: J Med Internet Res. 2022 Jan 11;24(1):e22122. doi: 10.2196/22122 (PMC8790687; doi:10.2196/22122)
Supplement: Multimedia Appendix 9 [file jmir_v24i1e22122_app9.pdf]

FREETEXT FEEDBACK ON REASONS FOR PREFERRED PLS

PSORIASIS

PSORIASIS: INFOGRAPHIC PREFERENCE (1 OF 3)

- Informality of language is annoying [high-complexity text] and [medium-complexity text] come across as having bias
- I think a mixture of graphics and text works better to explain such a complex subject.
- Graphics are helpful in getting statistical information across. The [medium complexity] summary combined straight forward language with enough information.
- I ranked the [low-complexity] text summary 4th because, although it was short and easy to read, it did not give me the pertinent statistical data for which the conclusion was drawn.
- The [high-complexity text] was way too hard to understand what they were saying, too many big words. I liked the other ones better because I could understand what they were saying. Also the graphic one would catch my eye in a doctors office.
- Graphic summery...clear, concise and easy to understand. [high complexity] summary was quiet complex to understand. [medium-complexity] summery was good. [low-complexity] summary did not give much info.

PSORIASIS: INFOGRAPHIC PREFERENCE (2 OF 3)

- From easiest to hardest to understand
- Very easy to understand all summaries very basic questions
- Summaries without "numbers" looks more like an common article, not necessarily scientific. More "hard" data makes me feel that study was serious and that it's not a bogus.
- Super easy to read layout, graphic images are far easier to digest than lots of words and looks less dry. Big thumbs up for more info graphics in medical research please!
- The graphic summary allowed for little bias in the presentation of the facts but could have benefited from having the two groups side by side rather than top down for comparison. Beyond that [low-complexity] summary was too simple and sounded a bit condescending. [high-complexity] summary by contrast had too much scientific detail/phrasing for the general public whereas [medium-complexity] summary hit the balance nicely.
- I prefer the graphic summary because it's easy to read and uses simple language

PSORIASIS:
INFOGRAPHIC PREFERENCE (3 OF 3)

- The graphic summary is easier to follow/ visualise
- I am a visual learner so would always prefer graphics/charts etc. I get too distracted and can't take in information when there's too much to read and too much medical jargon etc hence the first passage was my least favourite. [medium-complexity text] was just right. Not too much reading, easy to understand and got the point across. [low-complexity text] was just slightly too short and didn't have enough information so even though I understood it I was left wondering "what else"
- The last one is the most understandable for general public meanwhile the first ones are probably to extensive

PSORIASIS: HIGH-COMPLEXITY TEXT PREFERENCE (1 OF 1)

- I really care about the details. I have a scientific background, I want to know the details - in fact I found myself referring back to the first (more detailed) written summary to clarify what the stats behind the statements were. I did like the graphical abstract, but I'd like it to come together with the more detailed version.
- Having had severe psoriasis for 30 years, I understand the terminology used in [the high complexity-text] and the graphic. [medium- and low-complexity summaries] are too brief for someone who suffers the illness but may be adequate for someone reading about medical research for other reasons.
- The [high-complexity] summary gave a good amount of detail into the background and requirements of the study. It was scientifically worded but clear to understand and gives the impression of knowledge and professionalism. The [medium-complexity summary] has a reduction in scientific background but is still full of information and has a professional feel. The poster is good as it clearly illustrates the dosage schedule but it does not give much information over background or aim of the study. The [low-complexity] summary was worst as it was very dumbed down. It have no real information on the study and as the reader I found the overly simple information underestimates the readers intelligence.

PSORIASIS: MEDIUM-COMPLEXITY TEXT PREFERENCE (1 OF 2)

- The [high-complexity] summary was difficult to read due to the terminology used. The [medium-complexity] summary was easy to read and was detailed enough. Whilst I normally like graphic summaries as this is a complex study it can become confusing in parts.
- As a non medically qualified person my preferred summary has enough information for me. If I was actively following research on psoriasis the first one would suit me better. I don't like the graphics as it is spread over too many pages although by reducing the size of the pictures and texts that would mostly address that issue. The graphics could be used to discuss with someone who doesn't read or write well. (I thought there were differant numbers in the 2 groups from the written summaries but in the graphics is says there is a differant number of participants in the 2 groups, maybe I have misunderstood).
- The [medium-complexity] summary had a good mix of stats and was easy to read and understand. The Graphic one could have been better if it had been shorter and summarised differently but didn't have the detail.

PSORIASIS: MEDIUM-COMPLEXITY TEXT PREFERENCE (2 OF 2)

- The [high-complexity] text was to scienctific jargon that alot of normal patients would struggle to understand. The [medium-complexity] text was well detailed and in a language that was easy to understand by anyone. The [low-complexity] text was to bland. The graohic was well detailed and alot easier to read than loads of text
- I understood my first choice easily. First time reading it. My second choice was too light on meaningful medical information. Strangely I usually prefer graphics explanations but found the graphic presentation too messy/complicated and not enough meaningful content.

PSORIASIS: LOW-COMPLEXITY TEXT PREFERENCE (1 OF 1)

- The graphic confused me a little and I prefer to read very simple text that is short and accessible.
- I found the [low-complexity text] most simple to read and adsorb, and I thought it was the simplest for 'most' people to read. The graphic summary was quite difficult to read and understand. Too much information can be misleading for people so a simple explanation in language that is easily understood is better. Also some medical terms may need an explanation 'some' people may not know for example what a 'placebo' is. Thank you.
- Plain English, short and to the point, sometimes requiring diagrams are simpler to assimilate information in my opinion
- I felt that the [low-complexity] text summary was a lot more clear for myself (I don't have a science background). Everything was spelt out more clearly and was a lot easier to understand. The Graphic summary didn't mean much to someone like me as mentioned, who doesn't have a scientific background as images didn't relate to anything in my mind if this makes sense? It is easier to read a black/white text and have it explained. With images/graphs I don't feel that enough detail is included. All three of the text summaries were of the right length to keep me interested without loosing focus.

FREETEXT FEEDBACK ON REASONS FOR PREFERRED PLS

MULTIPLE SCLEROSIS

MULTIPLE SCLEROSIS: INFOGRAPHIC PREFERENCE (1 OF 3)

- Graphics was eye catching and easy to read, next had more detail, next appeared more academically written and the last had the least information
- I manage graphics better than text
- Easy to read and understandable, plain English
- Easiest to discuss and extrapolate information
- I found the graph easier to read and understand. All the information was there on one page. With the text summaries they had different information of different pages. Big words and a lot of text to read can deter people.
- number 4 had image related info which is easier to process number 2 was the best text to read number 3 seemed a little simple and number 1 was over complicated

MULTIPLE SCLEROSIS: INFOGRAPHIC PREFERENCE (2 OF 3)

- i think the most easy to understand is the graphical summary, although in my opinion there is too much text: graphical summaries in scientific articles are only about images, no text. About the text summaries, I believe there lacked information in all of them if the intention is that the reader is able to discuss the text with a doctor. I all cases, i think the information provided is somehow not accurate enough (e.g. "a type of white blood cell that play an important role in MS", i would prefer to say which is their importance, not just to say that they are important).
- 1. easy to understand, not too lengthy to read. nice graphics 2. enough info with numbers, but not too many to confuse you 3. not enough numbers - for all the reader knows, the study was conducted on a very small amount of people 4. too many numbers and scientific words/abbrevs. may get confusing
- the laaf one if by far the best and easiest to understand!
- Prefer the graphic one but also liked the detail in the first one. 2nd and 3rd were poorly written.

MULTIPLE SCLEROSIS: INFOGRAPHIC PREFERENCE (3 OF 3)

- Pictorial contained all the info of the medical summary number 1 but was much easier to quickly identify the main headlines
- The last one caught my attention better looked easier to read
- I prefer a visual presentation, rather than just words.
- I was really surprised at how accessible the graphic summary was. I am an academic and used to reading papers but it was quite hard to understand why it was said there were two studies as they appeared to be two trials of the same study with different medication. In the graphic summary this was much clearer.
- The larger ones have too much detail probably too difficult to understand for general public. The last one is the best since it has enough information which is at the same time easy to read. The penultimate one is good too
- would have given text [medium and low complexity] a rank of 1.....bitbeasy to read& understand graphic would remain2 medical txt 4......to much medical terminology /jargon used for lay person to understand

MULTIPLE SCLEROSIS: HIGH-COMPLEXITY TEXT PREFERENCE (1 OF 1)

- ease of understanding
- I personally prefer more information as provided in the first summary and for me the graphic summary slightly confuses me. The only difficulty I had was with the font chosen for the written summary/s, I have some problems with optic neuritis and the rounder fonts are easier for me to read.
- It could have been in any order because they were all easy to read.
- As most research is presented in the style 1 manner, I've become more accustomed to it and now find it frustrating when the detail is omitted in other sources of information. I think option 4 vs option
- Easy to read, less technical for someone with no medical background
- The graphic summary is too"busy" and all of the others are too simplistic - no reference to evidence

MULTIPLE SCLEROSIS: MEDIUM-COMPLEXITY TEXT PREFERENCE (1 OF 1)

- Too much or too little detail just raises more questions than it answers.

MULTIPLE SCLEROSIS: LOW-COMPLEXITY TEXT PREFERENCE (1 OF 1)

- Based on the ease to read and understand
- ok

FREETEXT FEEDBACK ON REASONS FOR PREFERRED PLS

RHEUMATOID ARTHRITIS

RHEUMATOID ARTHRITIS: INFOGRAPHIC PREFERENCE (1 OF 5)

- Graphics tend to be easier to understand.
- Graphics are always the best way to get a message across to all abilities
- I chose graphic as best as it gives the same info clearly and holds my attention. The 1st in relation to other examples was too technical. I had to keep rereading although I did understand.
- I ranked them in that order. Reason for it I found that people would under stand it better. Educated or not
- Need to know basis. Don't need to be baffled with science - we take enough drugs to fill a pharmacy.
- Graphics are much easier to understand
- Too much medical 'blurb' is confusing..causing me to skim and for both reasons not understand info. Although the second ,'medium info', was the best I found the graphics an absolutely brilliant idea. My A Level was in English, but having RA and fibromyalgia causes concentration issues..the graphics was so much easier to understand.
- The information albeit the same got easier to understand in each example.

RHEUMATOID ARTHRITIS: INFOGRAPHIC PREFERENCE (2 OF 5)

- Infographics show clarity - text only is much more difficult to read and understand- although I am a RA patient I am also a senior Research Nurse who is currently studying an MSc in clinical research and therefore my understanding is probably above a regular patient! However the use of info graphics I believe are much better understood especially if the person has no experience of reading scientific papers.
- I found [graphic] the easiest to digest and understand as i haven’t done any prior research into this drug.
- easier to understand
- The visual one was easy to follow, I didn’t like the [low-complexity] text, it was too simply written
- The graphics, visual colours and columns made it easier to understand.
- Graphics and bullet points always better than straight text
- Understood at a glance what study was about and the out come
- First was really difficult to understand. I liked the graphic one it was really clear. Don't know whether it mattered that that was the last one so already knew some things about the study.

RHEUMATOID ARTHRITIS: INFOGRAPHIC PREFERENCE (3 OF 5)

- The graphical poster is more eye catching and therefore easier to retain the infirmation. The [high-complexity text] is more medical jargon and therefore not really easy to understand as the patient.
- Graphs and visuals were easier to understand. The layout allowed for comparisons and taking in information easily.
- like the graphic easyer to understand
- The graphic summary made it much easier to understand, but did require a little more care in reading it. The third summary didn't give enough detail and the first was hard work!
- The first requires greater concentration and a previous experience of medical terminology - i.e. AE - not all would know that this means Adverse Event. The [medium-complexity] text used the same terminology but was less complex somehow. I thought the [low-complexity] text summary had been simplified to the extent that it lost some meaning, and the fourth graphic summary was accessible almost at a glance. Personally I would prefer to have access to both [high- and medium-complexity], the [medium] would 'fill in the gaps' and provide the detail that the summary by its very nature could not.

RHEUMATOID ARTHRITIS: INFOGRAPHIC PREFERENCE (4 OF 5)

- The final summary with the graphics enables the reader to see easily what the outcomes of the test were. Many people with RA have eye issues too so a bright and colour full page certainly helps us. That’s why i ranked this first. My second place choice was for the intermediate detailed summary. It had enough information to inform but not bamboozle. Third place was for the very detailed summary. I would rather know more than less information. And finally, the paragraph was just not detailed enough for me.
- The [medium-complexity] one, was much easier to read
- I like the graph because it's easy to read and grasp what's being said. I then would go for a more detailed report for more information.
- I found the graphs/charts put points across more easily
- I love reading graphs and am dyslexic, so text is harder for me and much slower to absorb. The first text contained too much detail which distracted my attention and focus from the key message being told. The second is in plain English and easily gets across the main messages.
- I have a photographic memory so visual is much easier and quicker otherwise I just scan it.

RHEUMATOID ARTHRITIS: INFOGRAPHIC PREFERENCE (5 OF 5)

- graphics was the better one and looked more informative.
- The first summary used far too much techinical information without lay explanation. the infographic is a far easier way to understand the findings
- I appreciated the visual aspect and "bullet point" style of the graphic summary, which really helped to understand the content and message of the summary. The writing style of the 2nd plain text was by far the easiest to read for me, with the 1st text being slightly too scientific and the 3rd feeling a bit oversimplified.

RHEUMATOID ARTHRITIS: HIGH-COMPLEXITY TEXT PREFERENCE (1 OF 4)

- Is much clearer
- Because the information provided was more detailed and I felt provided more information that, if so inclined, I could then further research into.
- Because I have a masters degree in biomedical science I'm used to reading journal articles and prefer words over graphics.
- It was easy to read probably and get all the information from the graphic definitely make all the details clear
- As someone who comes from a scientific background, I found the 1st style of summary for me more informative. The infographic I feel was great but could have been presented in a better way, some people may not understand the flow charts and lack of text at some parts but would probably be more interested at looking at an image with pictures and not just a wall of text.
- I like to be given as much detail as possible, so prefer the two summaries that give more factual information.

RHEUMATOID ARTHRITIS: HIGH-COMPLEXITY TEXT PREFERENCE (2 OF 4)

- I am used to reading clinical studies as I was a midwife and a health visitor. I found it helped me to understand more about the trial by reading the first study. I appreciate that for many patients that this could be too much information and could be more difficult to understand clearly. I think to appeal to the majority of patients the graphic summary was very clear and easy to understand and gave the main details you would need to know without all of the complicated medical terms. I think that the graphical summary was eye catching and it would make me look at it more than a page of writing.
- I liked the scientific way the first summary was presented and I liked the poster version too. I thought the other two weren't necessarily scientific enough
- Preferred writing styles and layout of information.
- I personally hate dumbed down items. I hate the way radio news is given as if it's for a child. Difficult subjects should be explained, not turned into a fairy story. I have read a lot of medical research (nursing degree) so know everyone would not like the first summary but i do. I would say for most people the second summary is a good compromise . I just hate picture posters with lots of graphics and no detail...again stuff for 5 year olds. I'm sick, not stupid.

RHEUMATOID ARTHRITIS: HIGH-COMPLEXITY TEXT PREFERENCE (3 OF 4)

- The graph could be slightly misread and little tricky to see what text related to what graphic first time looking at it. I liked the more technical content of summary 1 and if there is a word I do not understand it would be easy to find out. Summary 2 was still good but it would cause me to dig for more technical info but great as a basic overview. Summary 3 quite basic but still provides an overview. It totally depends what the summary target audience is and whether it is just an opening update of the topic for further discussion.
- I prefer the first simply because that is what i am used to reading when i read on line research throgh google scholar. The 2nd and 3rd were too diluted and over symplied while the graphic actually contained similar information to the first but in a very accessible form.
- The infographics in my opinion, where hard to follow and not very clear with ehat they were related too, with my befuddled brain, sometimes even the clearest things dont make sense though.
- Important to understand the detail and drop out rates
- I have studied physiology so the more detailed example was more suited to me but the last one was more to suit everyone

RHEUMATOID ARTHRITIS:
HIGH-COMPLEXITY TEXT PREFERENCE (4 OF 4)

- thats how i feel
- All easy to read but I think the first text page gave a better understanding of the survey and the graphics on the last survey were very easy to read and gave a good understanding of the survey in very easy to understand format.
- 1Contains more detailed info 2Detail plus ease of reading 3and 4 not enough detail.
- The first text summary seems to be for medics and the others were dumbing down the information for those who wouldn't understand the first. The info in the 4th was a good representation and easy to understand.

RHEUMATOID ARTHRITIS: MEDIUM-COMPLEXITY TEXT PREFERENCE (1 OF 5)

- Better understanding
- Clarity of info not, too much jargon but not too simplified. The graphic one is good as a poster.
- The 2nd summary concisely informed the reader about the trial and the drug including what it targeted (IL-6) and why and would be accessible to most people. The 1st summary may be a little too technical for some people although it was a concise summary of the trial. For me, the graphics were too long and there wasn't enough text/explanation, and the 3rd summary was too simple and there wasn't enough detail about what the drug treated and why.
- This is the order that I found them easiest to read.
- 2nd option had less medical terms, though it still had terms it was understandable. Graphic representation was great, I really liked the way it was set out and easily understandable. 1st option had way too much medical terminology and is slightly confusing, 4th option didn't have enough information for me, was too simplified
- Ease of understanding, more precise information

RHEUMATOID ARTHRITIS: MEDIUM-COMPLEXITY TEXT PREFERENCE (2 OF 5)

- I liked the info graphics however personally I like more detail about the study but would be happy with info graphic information
- The first summery example had to many facts and figures, the second one explained the results without too many figures, the third one was too basic and the poster style one was easy to read, had the information, but gave percentages rather than how many patients out of the group etc
- My preference would be for a combination of written information combined with graphics. I like enough information to feel informed but dislike too much abbreviation of terms.
- I work in a health comms agency so I prefer more detail and am used to understanding medical texts. I prefer text to graphics
- It is not easy to remember the order especially the first three. The graphic one omitted the higer dose of the drug side effects so I immediately mistrusted it. The others were ok. I dont like what looke dlike the playing down of side effects other than site reaction. How many people developed them in every class? What exactly? A casual remark about pnuemonia put the fear of God in me. This should have been presented more clearly.

RHEUMATOID ARTHRITIS: MEDIUM-COMPLEXITY TEXT PREFERENCE (3 OF 5)

- I would have put graphic 1st except it was not complete and had no conclusions.
- That’s the order in which I found them easiest to understand. For your information , I have a biology degree and used to work in immunology research , so may not be a typical patient!
- The first one gives an academic detail which I find fascinating but takes some concentration but not as much as four which is pretty uphill to understand. Two is excellent for a quick understanding and does compliment number one when read together. Number three is ok but I found I wanted much more depth.
- The third text was TOO simple. The graphics were good, but I got slightly lost about half way through. The first text, ranked fourth, was too detailed and too scientific.
- I find written info easier to assimilate than graphics. The first text required re reading at times to make it clear. The third text did not have sufficient detail. Therefore the second text was the most memorable.
- some people wouldn't understand the lengthy one with lots detail but would be good for those that could understand it

RHEUMATOID ARTHRITIS: MEDIUM-COMPLEXITY TEXT PREFERENCE (4 OF 5)

- The first text would be too complicated for a person who is not well educated and does not state the specific problem caused by the treatment - pneumonia. Written at academic level, the paper would need to state the specifics of what exactly or was the infection contracted. The second text is clear and serious in tone and has the important detail of the pneumonia infection. The third, showed more common language approach, but is patronizing. The fourth is good and very quick to understand. For most people taking in visual information is natural. The visual signs gave clues, for instance how many men in relation to women partook in the study. However, poster did not state anything about the third study group, nor where one could find more detailed information if one wished to do so.
- Clarity and content
- The second summary appealed to me as it had enough detail and was easierto comprehend. The graphic report was good to summarise findings but did not contain enough detail for me. The third summary did not contain enough detail and the first summary was very difficult to follow as it contained a large amount of technical phraseology
- Enough info but easy to understand balance

RHEUMATOID ARTHRITIS: MEDIUM-COMPLEXITY TEXT PREFERENCE (5 OF 5)

- 2nd text was clear but I felt needed more infrmation on the actual outcomes. The graphic summary was good and clear - easy to grasp - but where were the 12 month results?? (or did I miss them?). First text was overcompex and also, at times, ambiguous.
- I found the graphic summary very confusing; the third summary a little simplistic; the first a little too technical, so preferred summary two overall.

RHEUMATOID ARTHRITIS: LOW-COMPLEXITY TEXT PREFERENCE (1 OF 3)

- Ease of reading for [graphic] and [low]. [medium] was too busy and all over the place. [high] was just jargon
- The ease of reading the text and the flow. The pictures were to busy and needed more time to work out what it was actually saying
- I would prefer the graphic, if it had more details on it. I think that I was able to follow the graphic because I'd read the same description already three times. A combination of the last two samples would be ideal for me. Excellent study topic, thank you for your hard work
- How they improved my knowledge and how the text helped me retain the information.
- Simplifying language makes easier to understand
- I work verbally. Number three was clear and informative. Good use of language.
- I ranked from my understanding of the text
- For the balance of technical language to tabloid wording.

RHEUMATOID ARTHRITIS: LOW-COMPLEXITY TEXT PREFERENCE (2 OF 3)

- They are ranked that way as the easiest to understand is 1 down the the most confusing which I ranked 4.
- Balance of comprehensibility and comprehensiveness.
- In [high] and [medium]: language too technical, the layout of text is too dense - long paragraphs and lack of spacing too difficult to read.
- In my case I also have dyslexia so found it more easier to understand
- easiest to understand
- the [high complexity] one was just too "busy" it didn't make me want to read it in detail, [medium] and [low] were ok. The one with graphics might be ok if I were to see it on a doctors waiting room wall to study randomly
- Clear and informative in first choice; graphic too; too much info in first

RHEUMATOID ARTHRITIS:
LOW-COMPLEXITY TEXT PREFERENCE (3 OF 3)

- Easiest to understand the text
- I think the less there's to read is better for me due to concentration or lack of in my case
- Not really
